# Association of Lumbar Spine Radiographic Changes With Severity of Back Pain–Related Disability Among Middle-aged, Community-Dwelling Women

**DOI:** 10.1001/jamanetworkopen.2021.10715

**Published:** 2021-05-20

**Authors:** Lingxiao Chen, Romain S. Perera, Maja R. Radojčić, Paula R. Beckenkamp, Paulo H. Ferreira, Deborah J. Hart, Tim D. Spector, Nigel K. Arden, Manuela L. Ferreira

**Affiliations:** 1 Institute of Bone and Joint Research, The Kolling Institute, Northern Clinical School, Faculty of Medicine and Health, University of Sydney, Sydney, New South Wales, Australia; 2 Nuffield Department of Orthopaedics, Rheumatology and Musculoskeletal Sciences, University of Oxford, Oxford, United Kingdom; 3 Department of Allied Health Sciences, Faculty of Medicine, University of Colombo, Colombo, Sri Lanka; 4 Centre for Sport, Exercise and Osteoarthritis Research Versus Arthritis, University of Oxford, Oxford, United Kingdom; 5 School of Health Sciences, Faculty of Medicine and Health, University of Sydney, Sydney, New South Wales, Australia; 6 Department of Twin Research and Genetic Epidemiology, King’s College London, London, United Kingdom; 7 Medical Research Council Environmental Epidemiology Unit, University of Southampton, Southampton, United Kingdom

## Abstract

**Importance:**

Previous studies, using mostly cross-sectional data, provide conflicting evidence of an association between lumbar spine radiographic changes and the severity of back pain–related disability. Such conflicting evidence may be associated with widely unnecessary diagnostic imaging of the lumbar spine.

**Objective:**

To examine both cross-sectional and longitudinal associations between lumbar spine radiographic changes and the severity of back pain–related disability among middle-aged, community-dwelling women.

**Design, Setting, and Participants:**

This population-based prospective cohort study used data from the Chingford 1000 Women Study. Analyses included data collected from year 6 (1994-1996; physical activity was measured), year 9 (1997-1999; treated as baseline), and year 15 (2003-2005), with a total length of follow-up for longitudinal analyses of 6 years. Data were analyzed from April 17 to November 3, 2020.

**Exposures:**

Primary exposure was lumbar spine radiographic changes, defined using the Kellgren-Lawrence (K-L) grade. Secondary exposures were defined using presence of osteophytes and disc space narrowing. The composite score combined the number of lumbar spine segments with definite changes detected on radiographic images (ie, radiographic changes) (K-L grade ≥2, which means at least definite osteophyte and possible narrowing of disc space are present; osteophyte and disc space narrowing grade ≥1, which means at least mild or definite changes are present).

**Main Outcomes and Measures:**

Self-reported back pain–related disability measured in years 9 and 15 assessed by the St Thomas disability questionnaire.

**Results:**

Among 650 women (mean [SD] age, 61.3 [5.9] years) in cross-sectional analyses and 443 women (mean [SD] age, 60.6 [6.0] years) in longitudinal analyses, there was no evidence to support an association between higher number of lumbar segments with radiographic changes (K-L grade, osteophytes, and disc space narrowing) and more severe back pain–related disability (eg, cross-sectional analyses using the K-L grade; 1 segment vs 0 segment: adjusted odds ratio, 1.22 [95% CI, 0.76-1.96]). No interactions were found of an association between lumbar spine radiographic changes and the severity of back pain–specific disability with age, body mass index, or smoking status.

**Conclusions and Relevance:**

In this cohort of middle-aged, community-dwelling women, there was no evidence to support an association between a higher number of lumbar segments with radiographic changes (K-L grade, osteophytes, and disc space narrowing) and more severe back pain–related disability cross-sectionally or over time. These findings provide further evidence against routinely using diagnostic imaging of the lumbar spine.

## Key Points

**Question:**

Are lumbar spine radiographic changes associated with severity of back pain–related disability among middle-aged, community-dwelling women?

**Findings:**

In this population-based cohort study of women from the UK, there was no evidence to support an association between a higher number of lumbar segments with radiographic changes (Kellgren-Lawrence grade, osteophytes, and disc space narrowing) and more severe back pain–related disability in cross-sectional (650 women) or longitudinal (443 women) analyses.

**Meaning:**

The findings suggest that the changes detected on lumbar radiographs provide limited value for decision-making regarding back pain management in this population.

## Teaser

This cohort study uses data from the Chingford 1000 Women Study to examine both cross-sectional and longitudinal associations between lumbar spine radiographic changes and the severity of back pain–related disability among middle-aged, community-dwelling women.

## Introduction

Low back pain (LBP) is a highly prevalent condition in the general population worldwide and has been the leading cause of disability for nearly 3 decades, according to the Global Burden of Disease Study 2017.^1,2^ Among all musculoskeletal problems, LBP is also the most common reason for patients to seek primary care.^3^ Current guidelines for treatment of LBP do not recommend routinely using diagnostic imaging, except when patients either present with severe, progressive neurologic deficits or with signs or symptoms indicative of a serious or specific underlying condition (eg, fracture or cancer).^4,5,6^ Nonetheless, diagnostic imaging is still widely used in clinical practice for LBP, with a recent meta-analysis indicating that more than 15% of patients in primary care and approximately 25% in emergency care receive a referral for simple imaging (mainly radiograph).^7^ Moreover, nearly 10% of patients with LBP in primary care and emergency care also receive a referral for complex imaging (mainly computed tomography scan and magnetic resonance imaging).^7^ There has also been a 53% relative increase in referrals for complex imaging from 1995 to 2017, with no change observed during that period for the rate of referrals for simple imaging.^7^ Unnecessary diagnostic imaging not only wastes limited medical resources but is also associated with poorer health outcomes, such as iatrogenic disease from techniques that use ionizing radiation.^8^ In addition, patients who undergo unnecessary diagnostic imaging might be labeled with a pseudodisease, which may be associated with unnecessary subsequent interventions that may have adverse effects.^9^

Possible explanations for the unwarranted prevalence of imaging referrals for LBP are (1) the patient’s expectation that imaging results could provide valuable information on the cause and, consequently, the appropriate management of her or his condition and (2) the clinician’s desire to reassure the patient of the absence of any underlying pathologic condition.^10,11,12^ Previous studies have confirmed that imaging does not improve clinical outcomes for patients with LBP.^9,13^ However, the definition of normal or abnormal imaging diagnostic findings is still debatable. Currently, the presence of osteophytes and disc space narrowing are the most frequent changes detected on radiographs (hereafter referred to as radiographic changes) that may be indicative of spinal pathologic conditions, and the Kellgren-Lawrence (K-L) grade is the tool commonly used to assess the severity of osteoarthritis.^14^ A study including elderly women who lived in rural South Korea showed a positive association of the presence of osteophytes (grade ≥2), disc space narrowing (grade ≥2), and K-L grade (grade ≥2) with the severity of disability, measured by a validated Korean version of the Oswestry Disability Index.^15^ However, a study conducted in Sri Lanka including patients with LBP concluded that neither disc space narrowing nor the presence of osteophytes was associated with the severity of disability (also measured with the Modified Oswestry Disability Index).^16^ Both studies are cross-sectional and failed to adjust their analyses for important confounders, including smoking status, level of participation in physical activity, and medication use. Past studies have also failed to identify whether the number of affected lumbar segments is associated with the severity of back pain–related disability. Therefore, the role of radiographic findings as a potential prognostic factor of the clinical course of LBP is still unclear and needs to be fully explored in population-based cohort studies. Previous studies of radiographic changes in knee osteoarthritis have indicated that the presence of osteophytes may be used to diagnose the condition and that the presence of joint space narrowing may be used to assess both the diagnosis and the progression of osteoarthritis.^17,18^ This is still to be elucidated among patients with LBP.

The aim of this study was to examine both cross-sectional and longitudinal associations between lumbar radiographic changes and the severity of back pain–related disability among middle-aged, community-dwelling women using composite scores that combined the number of segments and type of changes in terms of K-L grade, disc space narrowing, and osteophytes. We hypothesized that a higher number of segments with lumbar radiographic changes would be associated with more severe back pain–related disability.

## Methods

### Study Design, Data Sources, and Study Population

From an age and sex register of a large practice of more than 11 000 patients in Chingford in east London, UK, all 1353 women in the age range of 45 to 64 years were invited to participate in a population study assessing musculoskeletal diseases. A total of 1003 women were examined between 1989 and 1991 (year 1; baseline visit for original cohort); 6 died, 66 had moved away, and 278 refused to participate or did not respond. All the women lived within 8 km (5 miles) of the general practice, and 98% of the women were white. Women from this general practice are similar to the UK general population in terms of weight, height, and body mass index (BMI). In the data analyses, and given their availability, we included data on physical activity collected in year 6 (1994-1996 [ie, prebaseline]) and imaging data, all other covariates, and the outcome for cross-sectional analyses collected in year 9 (1997-1999 [ie, baseline for our study]). The outcome for longitudinal analyses was obtained in year 15 (2003-2005). We followed the Strengthening the Reporting of Observational Studies in Epidemiology (STROBE) reporting guideline.^19,20^ The Waltham Forest and Redbridge local research ethics committee approved the study, and all participants provided written informed consent to participate in the study.

### Exposures

Lateral lumbar spine radiographs at year 9 were taken by 1 radiographer, centered on the L3 vertebra, with the participants in the left lateral recumbent position. A single trained observer (a rheumatologist) blinded to patient identity and chronologic order read all of the radiographs. Within-observer variation was assessed by test-retest analysis of 40 randomly selected radiographs from the study. Good within-observer reproducibility (κ = 0.78-0.89) was found.^21^ At each lumbar spine segment (L1-L2, L2-L3, L3-L4, and L4-L5), disc space narrowing and osteophytes (both anterior and posterior) were assessed through the semiquantitative method reported by Lane et al,^22^ with grade 0 corresponding to normal, grade 1 to mild narrowing and osteophytes, grade 2 to moderate narrowing and osteophytes, and grade 3 to severe narrowing and osteophytes. The Kellgren-Lawrence (K-L) grade was summarized as grade 0 indicating normal; grade 1 indicating doubtful narrowing of disc space and possible osteophytic lipping; grade 2 indicating definite osteophyte and possible narrowing of disc space; grade 3 indicating moderate multiple osteophytes, definite narrowing of disc space, some sclerosis, and possible deformity of bone contour; and grade 4 indicating large osteophytes, marked narrowing of disc space, severe sclerosis, and definite deformity of bone contour.

Considering that the number of lumbar spine segments with radiographic changes detected and the various types of radiographic changes might be associated with the results, we generated 3 composite scores: a K-L grade–based score, an osteophyte grade–based score, and a disc space narrowing grade–based score; at each segment, a binary exposure variable of 1 (K-L grade ≥2, which means at least definite osteophyte and possible narrowing of disc space are present; disc space narrowing and osteophyte grade ≥1, which means at least mild or definite changes are present) vs 0 (K-L grade 0 or 1; disc space narrowing and osteophyte grade 0) was used. The composite score was then calculated as the final L1-L2 score + L2-L3 score + L3-L4 score + L4-L5 score, with values ranging from 0 to 4 (where 0 indicates no lumbar spine segments with radiographic changes detected and 4 indicates 4 lumbar spine segments with radiographic changes detected). The K-L grade–based score was defined as the primary exposure. Osteophyte and disc space narrowing grade–based scores were set as secondary exposures.

### Outcomes

Back pain–related disability was assessed at year 9 and year 15 using a back pain questionnaire (St Thomas disability questionnaire), which correlated well with the Oswestry Disability Questionnaire (*r* = 0.77; *P* < .001).^23^ The outcome was defined by questions at 2 levels. At the first level, women were asked whether they had any back pain for at least 1 day at any time in the last 12 months. At the second level, those who answered yes to the first-level question were asked 8 questions related to the disability due to back pain (corresponding to the previous year’s status): walking around the house; standing for 15 minutes; getting up from a low chair; getting out of a bath; getting in and out of a car; going up and down stairs; putting on socks, stockings, or tights; and cutting toenails. Each question was summarized as grade 0 indicating no difficulty, grade 1 indicating difficult but possible, and grade 2 indicating impossible. We built a composite score based on the aforementioned 8 questions; values ranged from 0 to 16, with higher values corresponding to more severe disability. We assumed the composite score as 0 if women answered “no” to the first-level question. In case of missing data for any of the 8 questions, we kept the data if the women responded to at least 6 questions and calculated the composite score as [(total score)/(number of questions answered)] × 8.

### Covariates

Causal diagram through DAGitty, version 3.0^24^ was used to choose the minimal sufficient adjustment sets for estimating the total association of the exposure with the outcome.^25^ Age, BMI, smoking status, back pain status, bisphosphonate use, and physical activity were included in the final model (details in eFigure 1 in the Supplement). All covariates, except physical activity, which was measured in year 6, were measured in year 9.

### Statistical Analysis

Data were analyzed from April 17 to November 3, 2020. Owing to the skewed distribution of back pain–related disability (eFigure 2 in the Supplement), ordinal logistic regression, which holds a proportional odds assumption, was performed.^26^ Considering that physical activity was measured at a different time point (ie, year 6) compared with other covariates (ie, year 9) and with potential measurement error, we established a stepped modeling framework: step 1, unadjusted analyses; step 2, analyses adjusted for age, BMI, back pain status, bisphosphonate use status, and smoking status (additionally adjusted for year 9 back pain–related disability for the longitudinal analysis); and step 3, analyses further adjusted for physical activity.

Separate analyses were conducted for cross-sectional and longitudinal data. For the longitudinal analyses, data on lumbar spine radiographic changes collected in year 9 were treated as the exposure, and back pain–related disability data collected in year 15 were treated as the outcome. In addition to the confounders mentioned, data on back pain–related disability collected in year 9 were included in the longitudinal analysis as a strong prognostic factor to adjust.^27^ Based on the recommendation from *Modern Epidemiology*,^28^ the exposures were modeled as unordered categorical variables and trend test.^26^

The proportion of missing data in each covariate is provided in Table 1. Missing data were handled through multiple imputation, which holds a missing-at-random assumption.^26^ The assumption was graphically tested (eFigure 3 in the Supplement). No additional variables were used; all covariates in the minimal sufficient adjustment sets were used. Flexible additive models with 10 imputed data sets were used.^29^ We did not impute data for the exposure variables. The relative risk was presented as adjusted proportional odds ratios (ORs) with 95% CIs. Extensive sensitivity analyses were performed (eAppendix 1 in the Supplement). All statistical analyses were performed with rms, Hmisc, and tidyverse packages in R, version 3.6.2 (R Group for Statistical Computing). Details of the statistical methods are provided in eAppendix 2 in the Supplement.

**Table 1.** Baseline Characteristics of Study Participants

Characteristic	Participants, No. (%)					
	No. of segments^a^					Whole cohort
	0	1	2	3	4	
**Cross-sectional (n = 650)**						
No.	154	142	140	118	96	650
Age, mean (SD), y	59.7 (5.5)	60.3 (5.7)	60.8 (5.8)	62.8 (5.7)	64.5 (5.8)	61.3 (5.9)
Missing	1 (0.6)	2 (1.4)	4 (2.9)	2 (1.7)	1 (1.0)	10 (1.5)
Smoking status						
Never	105 (68.2)	84 (59.2)	89 (63.6)	71 (60.2)	61 (63.5)	410 (63.1)
Current	18 (11.7)	25 (17.6)	20 (14.3)	20 (16.9)	15 (15.6)	98 (15.1)
Ex-smoker	28 (18.2)	31 (21.8)	25 (17.9)	23 (19.5)	18 (18.8)	125 (19.2)
Missing	3 (1.9)	2 (1.4)	6 (4.3)	4 (3.4)	2 (2.1)	17 (2.6)
BMI, mean (SD)	26.6 (4.2)	26.8 (5.1)	27.2 (5.0)	26.9 (4.4)	27.6 (5.4)	27.0 (4.8)
Missing	1 (0.6)	3 (2.1)	4 (2.9)	4 (3.4)	1 (1.0)	13 (2.0)
Back pain status						
Yes	51 (33.1)	46 (32.4)	39 (27.9)	42 (35.6)	32 (33.3)	210 (32.3)
No	103 (66.9)	96 (67.6)	101 (72.1)	76 (64.4)	64 (66.7)	440 (67.7)
Bisphosphonate use status						
Yes	5 (3.2)	3 (2.1)	4 (2.9)	1 (0.8)	1 (1.0)	14 (2.2)
No	68 (44.2)	62 (43.7)	71 (50.7)	55 (46.6)	44 (45.8)	300 (46.2)
Missing	81 (52.6)	77 (54.2)	65 (46.4)	62 (52.5)	51 (53.1)	336 (51.7)
Physical activity						
Walking, km/wk						
<0.8	14 (9.1)	6 (4.2)	12 (8.6)	7 (5.9)	13 (13.5)	52 (8.0)
0.8 to <8.1	75 (48.7)	75 (52.8)	60 (42.9)	70 (59.3)	43 (44.8)	323 (49.7)
8.1 to <16.1	42 (27.3)	36 (25.4)	41 (29.3)	30 (25.4)	17 (17.7)	166 (25.5)
≥16.1	17 (11.0)	19 (13.4)	21 (15.0)	5 (4.2)	16 (16.7)	78 (12.0)
Missing	6 (3.9)	6 (4.2)	6 (4.3)	6 (5.1)	7 (7.3)	31 (4.8)
Job						
Sedentary	8 (5.2)	8 (5.6)	4 (2.9)	4 (3.4)	0	24 (3.7)
Sedentary plus occasional exercise	23 (14.9)	14 (9.9)	16 (11.4)	13 (11.0)	8 (8.3)	74 (11.4)
0.5 Sedentary plus 0.5 active (or active housework [eg, daily dusting or vacuuming])	88 (57.1)	77 (54.2)	87 (62.1)	73 (61.9)	59 (61.5)	384 (59.1)
Predominantly manual, active all day	27 (17.5)	30 (21.1)	22 (15.7)	17 (14.4)	15 (15.6)	111 (17.1)
Missing	8 (5.2)	13 (9.2)	11 (7.9)	11 (9.3)	14 (14.6)	57 (8.8)
Sport						
None	80 (51.9)	78 (54.9)	89 (63.6)	70 (59.3)	60 (62.5)	377 (58.0)
Golf, bowling, badminton, cycling, or swimming, 1 h/wk	22 (14.3)	26 (18.3)	15 (10.7)	15 (12.7)	8 (8.3)	86 (13.2)
2 h/wk of Golf, bowling, badminton, cycling, or swimming or 1 h of staying fit, aerobics, or squash	30 (19.5)	23 (16.2)	22 (15.7)	19 (16.1)	11 (11.5)	105 (16.2)
≥2 h/wk of Staying fit, aerobics, or squash	17 (11.0)	9 (6.3)	8 (5.7)	8 (6.8)	11 (11.5)	53 (8.2)
Missing	5 (3.2)	6 (4.2)	6 (4.3)	6 (5.1)	6 (6.3)	29 (4.5)
Disability, year 9, median (IQR)	0 (0.0-5.8)	0 (0.0-6.0)	0 (0.0-3.3)	0 (0.0-4.9)	0 (0.0-3.3)	0 (0.0-5.0)
**Longitudinal (n = 443)**						
No.	112	100	97	76	58	443
Age, mean (SD), y	59.1 (5.3)	59.5 (5.7)	60.5 (6.3)	62.6 (6.1)	62.8 (5.9)	60.6 (6.0)
Missing	1 (0.9)	2 (2.0)	3 (3.1)	0	0	6 (1.4)
Smoking status						
Never	77 (68.8)	63 (63.0)	64 (66.0)	44 (57.9)	38 (65.5)	286 (64.6)
Current	13 (11.6)	14 (14.0)	11 (11.3)	16 (21.1)	9 (15.5)	63 (14.2)
Ex-smoker	19 (17.0)	21 (21.0)	18 (18.6)	15 (19.7)	11 (19.0)	84 (19.0)
Missing	3 (2.7)	2 (2.0)	4 (4.1)	1 (1.3)	0	10 (2.3)
BMI, mean (SD)	26.4 (4.3)	26.7 (4.9)	27.2 (5.2)	26.5 (3.7)	28.3 (5.3)	26.9 (4.7)
Missing	1 (0.9)	3 (3.0)	3 (3.1)	2 (2.6)	0	9 (2.0)
Back pain status						
Yes	40 (35.7)	32 (32.0)	24 (24.7)	26 (34.2)	23 (39.7)	145 (32.7)
No	72 (64.3)	68 (68.0)	73 (75.3)	50 (65.8)	35 (60.3)	298 (67.3)
Bisphosphonate use status						
Yes	4 (3.6)	3 (3.0)	2 (2.1)	1 (1.3)	1 (1.7)	11 (2.5)
No	43 (38.4)	43 (43.0)	55 (56.7)	35 (46.1)	26 (44.8)	202 (45.6)
Missing	65 (58.0)	54 (54.0)	40 (41.2)	40 (52.6)	31 (53.4)	230 (51.9)
Physical activity						
Walking, km/wk						
<0.8	11 (9.8)	5 (5.0)	12 (12.4)	5 (6.6)	9 (15.5)	42 (9.5)
0.8 to <8.1	54 (48.2)	50 (50.0)	42 (43.3)	46 (60.5)	29 (50.0)	221 (49.9)
8.1 to <16.1	31 (27.7)	27 (27.0)	22 (22.7)	20 (26.3)	8 (13.8)	108 (24.4)
≥16.1	10 (8.9)	16 (16.0)	17 (17.5)	3 (3.9)	10 (17.2)	56 (12.6)
Missing	6 (5.4)	2 (2.0)	4 (4.1)	2 (2.6)	2 (3.5)	16 (3.6)
Job						
Sedentary	5 (4.5)	5 (5.0)	4 (4.1)	3 (3.9)	0	17 (3.8)
Sedentary plus occasional exercise	15 (13.4)	13 (13.0)	10 (10.3)	9 (11.8)	6 (10.3)	53 (12.0)
0.5 Sedentary plus 0.5 active (or active housework [eg, daily dusting or vacuuming])	65 (58.0)	55 (55.0)	62 (63.9)	49 (64.5)	37 (63.8)	268 (60.5)
Predominantly manual, active all day	21 (18.8)	24 (24.0)	14 (14.4)	11 (14.5)	10 (17.2)	80 (18.1)
Missing	6 (5.4)	3 (3.0)	7 (7.2)	4 (5.3)	5 (8.6)	25 (5.6)
Sport						
None	60 (53.6)	52 (52.0)	57 (58.8)	45 (59.2)	38 (65.5)	252 (56.9)
Golf, bowling, badminton, cycling, or swimming, 1 h/wk	14 (12.5)	21 (21.0)	15 (15.5)	8 (10.5)	4 (6.9)	62 (14.0)
2 h/wk of Previous or 1 h of staying fit, aerobics, or squash	22 (19.6)	19 (19.0)	16 (16.5)	17 (22.4)	6 (10.3)	80 (18.1)
≥2 h/wk of Staying fit, aerobics, or squash	11 (9.8)	6 (6.0)	4 (4.1)	4 (5.3)	8 (13.8)	33 (7.4)
Missing	5 (4.5)	2 (2.0)	5 (5.2)	2 (2.6)	2 (3.4)	16 (3.6)
Disability, year 15, median (IQR)	0 (0.0-5.0)	0 (0.0-5.1)	0 (0.0-4.0)	0 (0.0-2.5)	0 (0.0-4.8)	0 (0.0-4.8)

Abbreviations: BMI, body mass index (calculated as weight in kilograms divided by height in meters squared); IQR, interquartile range.

^a^ Number of segments of lumbar spine radiographic changes (based on Kellgren-Lawrence grade).

## Results

### Participant Characteristics

A total of 650 women (mean [SD] age, 61.3 [5.9] years) were included in cross-sectional analyses, and a total of 443 women (mean [SD] age, 60.6 [6.0] years) were included in longitudinal analyses (Table 1; Figure). Most study participants were classified as either never smokers or ex-smokers (Table 1). The median score of back pain–related disability was 0 (interquartile range, 0.0-5.0 in cross-sectional analyses and 0.0-4.8 in longitudinal analyses) in both cross-sectional and longitudinal analyses. The distribution of each lumbar spine radiographic change at each lumbar spine segment is listed in eTable 1 in the Supplement. Redundancy analyses were performed to assess whether 1 exposure could be estimated from any 2 other exposures, at each lumbar spine segment.^26^ No exposure was redundant (eTable 2 in the Supplement).

**Figure.**
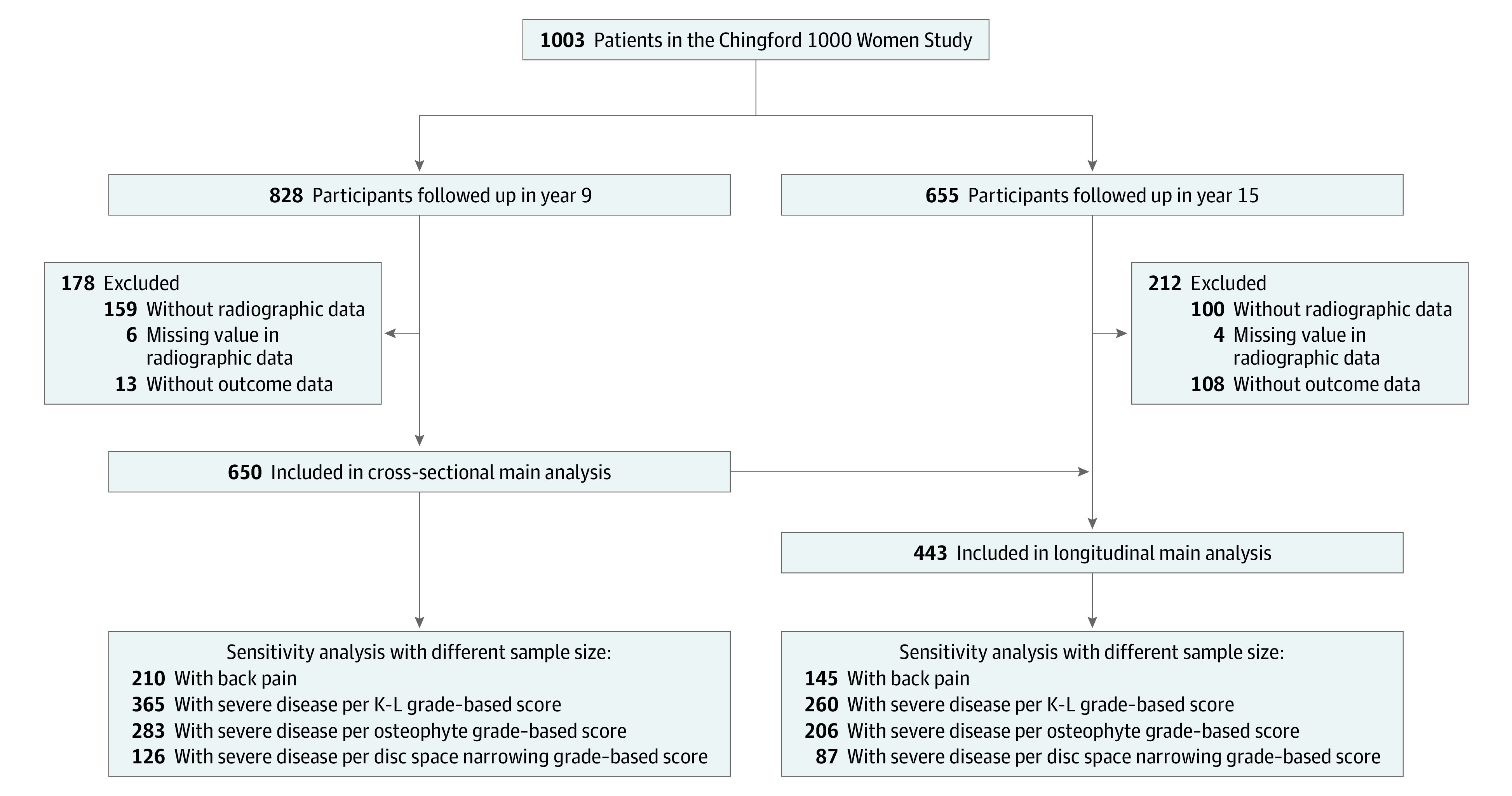
Flowchart of Study Participants A total of 95.0% women (421 of 443) in the longitudinal analysis are also in the cross-sectional analysis. Besides all data mentioned in the flowchart, we also collected the data from year 6 (1994-1996) because it was when 1 necessary covariate (ie, physical activity) was measured. K-L indicates Kellgren-Lawrence.

### K-L Grade–Based Score

Using a multivariable ordinal logistic regression model, we found that women who had 1 or more segments with lumbar spine radiographic K-L grade–based changes were not statistically more likely to report more disability compared with women with no observed changes in both cross-sectional (eg, 1 segment vs 0 segments; step 2 model; OR, 1.22 [95% CI, 0.76-1.96]) and longitudinal analyses (Table 2). When further adjustment was made for physical activity, the results were similar (OR, 1.19 [95% CI, 0.73-1.93]). No evidence was found to support a linear or nonlinear trend between number of segments with lumbar spine radiographic changes and the severity of back pain–related disability.

**Table 2.** Association Between K-L Grade–Based Score and Severity of Back Pain–Related Disability

Variable	K-L grade–based score, OR (95% CI)					*P* value for trend	
	0 Segments	1 Segment	2 Segments	3 Segments	4 Segments	Linear model	Nonlinear model
**Cross-sectional, year 9 (n = 650)**							
Women, No. (%)	154 (23.7)	142 (21.8)	140 (21.5)	118 (18.2)	96 (14.8)	NA	NA
Unadjusted	1 [Reference]	1.19 (0.75-1.89)	0.84 (0.52-1.36)	0.90 (0.54-1.49)	0.81 (0.47-1.38)	.24	.78
Multivariable adjusted^a^	1 [Reference]	1.22 (0.76-1.96)	0.84 (0.51-1.38)	0.92 (0.54-1.56)	0.89 (0.50-1.57)	.39	.91
Further adjusted for physical activity	1 [Reference]	1.19 (0.73-1.93)	0.81 (0.49-1.35)	0.87 (0.51-1.49)	0.85 (0.48-1.50)	.30	.96
**Longitudinal, year 15 (n = 443)**							
Women, No. (%)	112 (25.3)	100 (22.6)	97 (21.9)	76 (17.2)	58 (13.1)	NA	NA
Unadjusted	1 [Reference]	1.22 (0.70-2.11)	0.95 (0.53-1.68)	0.77 (0.41-1.44)	1.05 (0.55-2.02)	.57	.92
Multivariable adjusted^b^	1 [Reference]	1.06 (0.57-1.96)	0.94 (0.50-1.76)	0.69 (0.34-1.38)	0.83 (0.40-1.72)	.20	.79
Further adjusted for physical activity	1 [Reference]	1.08 (0.58-2.02)	0.91 (0.48-1.72)	0.67 (0.33-1.38)	0.80 (0.38-1.68)	.17	.80

Abbreviations: K-L, Kellgren-Lawrence; NA, not applicable; OR, odds ratio.

^a^ Adjusted for age, body mass index, smoking status, back pain status, and bisphosphonate use.

^b^ Adjusted for age, body mass index, smoking status, back pain status, bisphosphonate use, and year 9 back pain–related disability.

### Osteophyte Grade–Based Score and Disc Space Narrowing Grade–Based Score

For osteophyte grade–based score, no statistically significant association was found between the number of lumbar segments with radiographic changes and the severity of back pain–related disability in both cross-sectional (eg, 1 segment vs 0 segment; step 2 model; OR, 0.83 [95% CI, 0.57-1.22]) and longitudinal analyses (Table 3). Similar results were observed when further adjustments were made in the models to account for participation in physical activity in both cross-sectional and longitudinal analyses. In the longitudinal analysis, a greater number of affected segments were linearly associated with less severe back pain–related disability (step 2 model).

**Table 3.** Association Between Osteophyte Grade–Based Score and the Severity of Back Pain–Related Disability

Variable	Osteophyte grade–based score, OR (95% CI)					*P* value for trend	
	0 Segments	1 Segment	2 Segments	3 Segments	4 Segments	Linear model	Nonlinear model
**Cross-sectional, year 9 (n = 650)**							
Women, No. (%)	258 (39.7)	226 (34.8)	102 (15.7)	44 (6.8)	20 (3.1)	NA	NA
Unadjusted	1 [Reference]	0.80 (0.55-1.16)	0.81 (0.50-1.32)	0.53 (0.25-1.12)	0.81 (0.31-2.14)	.12	.59
Multivariable adjusted^a^	1 [Reference]	0.83 (0.57-1.22)	0.78 (0.47-1.30)	0.58 (0.27-1.26)	1.03 (0.37-2.85)	.25	.42
Further adjusted for physical activity	1 [Reference]	0.82 (0.56-1.21)	0.79 (0.47-1.32)	0.60 (0.28-1.31)	0.98 (0.35-2.73)	.26	.46
**Longitudinal, year 15 (n = 443)**							
Women, No. (%)	192 (43.3)	157 (35.4)	67 (15.1)	19 (4.3)	8 (1.8)	NA	NA
Unadjusted	1 [Reference]	0.71 (0.46-1.10)	0.60 (0.33-1.08)	0.41 (0.13-1.27)	0.49 (0.10-2.40)	.02	.65
Multivariable adjusted^b^	1 [Reference]	0.76 (0.47-1.24)	0.53 (0.28-1.02)	0.49 (0.14-1.70)	0.31 (0.06-1.72)	.01	.75
Further adjusted for physical activity	1 [Reference]	0.76 (0.46-1.24)	0.52 (0.27-1.03)	0.50 (0.14-1.74)	0.33 (0.06-1.79)	.01	.68

Abbreviations: NA, not applicable; OR, odds ratio.

^a^ Adjusted for age, body mass index, smoking status, back pain status, and bisphosphonate use.

^b^ Adjusted for age, body mass index, smoking status, back pain status, bisphosphonate use, and year 9 back pain–related disability.

For the disc space narrowing grade–based score, no statistically significant association was observed between the number of lumbar segments with radiographic changes and the severity of back pain–related disability in both cross-sectional (eg, 1 segment vs 0 segment; step 2 model; OR, 1.43 [95% CI, 0.78-2.61]) and longitudinal analyses (Table 4). Results were similar when we further adjusted for physical activity (OR, 1.41 [95% CI, 0.77-2.60]). For the cross-sectional analyses, a higher number of segments of lumbar spine radiographic characteristics were nonlinearly associated with less severe back pain–related disability (step 2 model).

**Table 4.** Association Between Disc Space Narrowing Grade–Based Score and the Severity of Back Pain–Related Disability

Variable	Disc space narrowing grade–based score, OR (95% CI)					*P* value for trend	
	0 Segments	1 Segment	2 Segments	3 Segments	4 Segments	Linear model	Nonlinear model
**Cross-sectional, year 9 (n = 650)**							
Women, No. (%)	100 (15.4)	107 (16.5)	147 (22.6)	131 (20.2)	165 (25.4)	NA	NA
Unadjusted	1 [Reference]	1.33 (0.74-2.37)	1.30 (0.75-2.24)	1.33 (0.77-2.31)	0.98 (0.57-1.69)	.75	.11
Multivariable adjusted^a^	1 [Reference]	1.43 (0.78-2.61)	1.56 (0.88-2.76)	1.44 (0.81-2.57)	1.07 (0.60-1.92)	.94	.04
Further adjusted for physical activity	1 [Reference]	1.41 (0.77-2.60)	1.56 (0.87-2.77)	1.45 (0.81-2.59)	1.04 (0.57-1.87)	.86	.03
**Longitudinal, year 15 (n = 443)**							
Women, No. (%)	70 (15.8)	84 (19.0)	102 (23.0)	88 (19.9)	99 (22.3)	NA	NA
Unadjusted	1 [Reference]	0.91 (0.47-1.75)	0.78 (0.41-1.49)	1.07 (0.56-2.05)	1.24 (0.67-2.29)	.34	.25
Multivariable adjusted^b^	1 [Reference]	0.72 (0.34-1.53)	0.74 (0.36-1.52)	1.06 (0.52-2.20)	1.26 (0.62-2.57)	.18	.18
Further adjusted for physical activity	1 [Reference]	0.68 (0.31-1.48)	0.67 (0.31-1.43)	1.12 (0.53-2.34)	1.33 (0.63-2.80)	.13	.12

Abbreviations: NA, not applicable; OR, odds ratio.

^a^ Adjusted for age, body mass index, smoking status, back pain status, and bisphosphonate use.

^b^ Adjusted for age, body mass index, smoking status, back pain status, bisphosphonate use, and year 9 back pain–related disability.

### Exploratory and Sensitivity Analyses

We did not find interactions with age, BMI, or smoking status (eTable 3 in the Supplement) between lumbar spine radiographic changes and the severity of back pain–related disability. Overall, our results remained similar under extensive sensitivity analyses (eAppendices 3-8 and eTables 4-25 in the Supplement). All E-values are listed in eTable 26 in the Supplement.

## Discussion

### Key Results

In this cohort of middle-aged women from Chingford in east London, UK, no evidence was found to support an association between a higher number of segments with lumbar radiographic changes (K-L grade, osteophyte, and disc space narrowing) and more severe back pain–related disability. Our results remained unchanged after including potential interactions with important confounders, such as age, BMI, and smoking status, and after extensive sensitivity analyses.

### Comparison With Previous Studies

For K-L results, our findings contradict those of Lee et al,^15^ who found that K-L grades were significantly associated with the Oswestry Disability Index. The main reason for such a discrepancy in results may be the design features of the study by Lee et al,^15^ which only included cross-sectional analyses with insufficient adjustment for important confounders (eg, physical activity, smoking status, and BMI). For osteophyte results, the results from the cross-sectional analyses are consistent with a previous cross-sectional study by Perera et al,^16^ who identified that the presence of osteophytes on radiographs was not associated with physical disability measured with the Oswestry Disability Index. However, results from our longitudinal analyses indicated that a greater number of affected segments were linearly associated with less severe back pain–related disability. One possible explanation is the biomechanical stability provided by spinal osteophytes, which have been proven to increase spinal resistance in compression.^30^ Another explanation is that the results simply reflect large numbers of analyses completed, which need future studies to verify. For disc space narrowing results, our results are similar to those of Perera et al^16^ but different than the findings of Lee et al.^15^ Such differences could also be associated with the methodological limitations already described in the study conducted by Lee et al.^15^ Overall, the prevalence of lumbar spine radiographic findings in our study is similar to that of previous studies that indicated that many imaging-based spinal radiographic changes are likely part of normal, asymptomatic aging.^31^

### Strengths and Limitations

Our study has several strengths. To our knowledge, this is the first study to create a composite score that reflects the overall association of lumbar spine radiographic changes (ie, number of affected segments and severity of changes) with the severity of back pain–related disability. We used population-based data that contain a long-term follow-up with good recruitment and retention rates, and multiple potential confounders were measured. We also overcame some methodological limitations from previous studies; we included a cohort study design and incorporated a systematic way to select and control confounders, we have repeated measures of back pain–related disability that allow us to adjust for baseline disability, we assessed the potential interaction term, and we performed extensive sensitivity analyses to evaluate the robustness of the results.

Limitations also need to be considered. First, the Chingford 1000 Women Study included middle-aged women in a specific area of the UK. We must exercise caution when generalizing the results to men, other age groups, other racial/ethnic groups, or other countries. Second, as with most studies, there is the potential for residual confounding (eg, participation in physical activity was measured 3 years before baseline). Third, the labeling of the images may have introduced potential bias in our results, given that there was only 1 observer and that lumbar spine levels were decided by the clinical experience. Fourth, owing to data unavailability, we could not establish whether there was any association between other radiologic changes, including spondylolisthesis or vertebral body height (ie, osteoporotic fractures), and severity of functional limitation. Fifth, although our outcome correlated well with the Oswestry Disability Questionnaire, it lacked strict validation. Sixth, although we aimed to focus on LBP–related disability, we only had a back pain variable, which might be slightly different from LBP.

### Implications for Practice and Research

Clinicians may use the results of this study to educate patients and their colleagues that lumbar radiographic findings cannot provide prognostic information on back pain–related disability, further adding to the evidence supporting the urge to reduce unnecessary imaging referrals. Future studies should include participants of both sexes and larger sample sizes and should include multiple centers to increase external validity. The association between the findings of complex imaging (eg, computed tomography scans, magnetic resonance imaging, or nuclear bone scans) and symptom severity in people with LBP needs to be further explored, considering the increasing use of such imaging.

## Conclusions

In this cohort of middle-aged, community-dwelling women, there was no evidence to support an association between a higher number of lumbar segments with radiographic changes (K-L grade, osteophytes, and disc space narrowing) and more severe back pain–related disability cross-sectionally or over time. The findings suggest that the changes detected on lumbar radiographs provide limited value for decision-making regarding back pain management in this population.

## Notes

Supplement. **eFigure 1.** Causal Diagram **eFigure 2.** Histogram of Outcome Distribution **eFigure 3.** Missing Data Pattern **eAppendix 1.** Methods for Exploratory and Sensitivity Analyses **eAppendix 2.** Details of Statistical Methods **eTable 1.** Distribution of Lumbar Spine Radiographic Changes at Each Lumbar Spine Segment **eTable 2.** Redundancy Analysis of Exposures **eTable 3.** Interaction with Age, BMI, or Smoking Status **eAppendix 3.** Sensitivity Analyses Results: Change on the Cut-Off Points of Exposures **eTable 4.** Kellgren-Lawrence Grade–Based Score **eTable 5.** Osteophytes Grade–Based Score **eTable 6.** Disc Space Narrowing Grade–Based Score **eAppendix 4.** Sensitivity Analyses Results: Total Original Score of Exposures **eTable 7.** Kellgren-Lawrence Grade **eTable 8.** Osteophytes Grade **eTable 9.** Disc Space Narrowing Grade **eAppendix 5.** Sensitivity Analyses Results: Disease Severity **eTable 10.** Kellgren-Lawrence Grade–Based Score **eTable 11.** Osteophytes Grade–Based Score **eTable 12.** Disc Space Narrowing Grade–Based Score **eAppendix 6.** Sensitivity Analyses Results: Restricting to Women With Back Pain **eTable 13.** Kellgren-Lawrence Grade–Based Score **eTable 14.** Osteophytes Grade–Based Score **eTable 15.** Disc Space Narrowing Grade–Based Score **eAppendix 7.** Sensitivity Analyses Results: Changing the Model to cloglog Link Function **eTable 16.** Kellgren-Lawrence Grade–Based Score **eTable 17.** Osteophytes Grade–Based Score **eTable 18.** Disc Space Narrowing Grade–Based Score **eAppendix 8.** Sensitivity Analyses Results: Change the Model to Linear Regression **eTable 19.** Kellgren-Lawrence Grade–Based Score **eTable 20.** Osteophytes Grade–Based Score **eTable 21.** Disc Space Narrowing Grade–Based Score **eTable 22.** Additionally Adjusted for Pain Medication and Depression **eTable 23.** Kellgren-Lawrence Grade–Based Score **eTable 24.** Osteophytes Grade–Based Score **eTable 25.** Disc Space Narrowing Grade–Based Score **eTable 26.** E-Value
