# Supplementary material for: Association of Lumbar Spine Radiographic Changes With Severity of Back Pain–Related Disability Among Middle-aged, Community-Dwelling Women
Source: JAMA Netw Open. 2021 May 20;4(5):e2110715. doi: 10.1001/jamanetworkopen.2021.10715 (PMC8138688; doi:10.1001/jamanetworkopen.2021.10715)
Supplement: Supplement. — eFigure 1. Causal Diagram eFigure 2. Histogram of Outcome Distribution eFigure 3. Missing Data Pattern eAppendix 1. Methods for Exploratory and Sensitivity Analyses eAppendix 2. Details of Statistical Methods eTable 1. Distribution of Lumbar Spine Radiographic Changes at Each Lumbar Spine Segment eTable 2. Redundancy Analysis of Exposures eTable 3. Interaction with Age, BMI, or Smoking Status eAppendix 3. Sensitivity Analyses Results: Change on the Cut-Off Points of Exposures eTable 4. Kellgren-Lawrence Grade–Based Score eTable 5. Osteophytes Grade–Based Score eTable 6. Disc Space Narrowing Grade–Based Score eAppendix 4. Sensitivity Analyses Results: Total Original Score of Exposures eTable 7. Kellgren-Lawrence Grade eTable 8. Osteophytes Grade eTable 9. Disc Space Narrowing Grade eAppendix 5. Sensitivity Analyses Results: Disease Severity eTable 10. Kellgren-Lawrence Grade–Based Score eTable 11. Osteophytes Grade–Based Score eTable 12. Disc Space Narrowing Grade–Based Score eAppendix 6. Sensitivity Analyses Results: Restricting to Women With Back Pain eTable 13. Kellgren-Lawrence Grade–Based Score eTable 14. Osteophytes Grade–Based Score eTable 15. Disc Space Narrowing Grade–Based Score eAppendix 7. Sensitivity Analyses Results: Changing the Model to cloglog Link Function eTable 16. Kellgren-Lawrence Grade–Based Score eTable 17. Osteophytes Grade–Based Score eTable 18. Disc Space Narrowing Grade–Based Score eAppendix 8. Sensitivity Analyses Results: Change the Model to Linear Regression eTable 19. Kellgren-Lawrence Grade–Based Score eTable 20. Osteophytes Grade–Based Score eTable 21. Disc Space Narrowing Grade–Based Score eTable 22. Additionally Adjusted for Pain Medication and Depression eTable 23. Kellgren-Lawrence Grade–Based Score eTable 24. Osteophytes Grade–Based Score eTable 25. Disc Space Narrowing Grade–Based Score eTable 26. E-Value [file jamanetwopen-e2110715-s001.pdf]

Supplementary Online Content

Chen L, Perera RS, Radojčić MR, et al. Association of lumbar spine radiographic changes with severity of back pain–related disability among middle-aged, community-dwelling women. *JAMA Netw Open.* 2021;4(5):e2110715. doi:10.1001/jamanetworkopen.2021.10715

eFigure 1. Causal Diagram
eFigure 2. Histogram of Outcome Distribution
eFigure 3. Missing Data Pattern
eAppendix 1. Methods for Exploratory and Sensitivity Analyses
eAppendix 2. Details of Statistical Methods
eTable 1. Distribution of Lumbar Spine Radiographic Changes at Each Lumbar Spine Segment
eTable 2. Redundancy Analysis of Exposures
eTable 3. Interaction with Age, BMI, or Smoking Status
eAppendix 3. Sensitivity Analyses Results: Change on the Cut-Off Points of Exposures
eTable 4. Kellgren-Lawrence Grade–Based Score
eTable 5. Osteophytes Grade–Based Score
eTable 6. Disc Space Narrowing Grade–Based Score
eAppendix 4. Sensitivity Analyses Results: Total Original Score of Exposures
eTable 7. Kellgren-Lawrence Grade
eTable 8. Osteophytes Grade
eTable 9. Disc Space Narrowing Grade
eAppendix 5. Sensitivity Analyses Results: Disease Severity
eTable 10. Kellgren-Lawrence Grade–Based Score
eTable 11. Osteophytes Grade–Based Score
eTable 12. Disc Space Narrowing Grade–Based Score
eAppendix 6. Sensitivity Analyses Results: Restricting to Women With Back Pain
eTable 13. Kellgren-Lawrence Grade–Based Score
eTable 14. Osteophytes Grade–Based Score
eTable 15. Disc Space Narrowing Grade–Based Score
eAppendix 7. Sensitivity Analyses Results: Changing the Model to cloglog Link Function
eTable 16. Kellgren-Lawrence Grade–Based Score
eTable 17. Osteophytes Grade–Based Score
eTable 18. Disc Space Narrowing Grade–Based Score
eAppendix 8. Sensitivity Analyses Results: Change the Model to Linear Regression
eTable 19. Kellgren-Lawrence Grade–Based Score
eTable 20. Osteophytes Grade–Based Score
eTable 21. Disc Space Narrowing Grade–Based Score
eTable 22. Additionally Adjusted for Pain Medication and Depression
eTable 23. Kellgren-Lawrence Grade–Based Score
eTable 24. Osteophytes Grade–Based Score
eTable 25. Disc Space Narrowing Grade–Based Score
eTable 26. E-Value

© 2021 Chen L et al. *JAMA Network Open.*

This supplementary material has been provided by the authors to give readers additional information about their work.

© 2021 Chen L et al. *JAMA Network Open.*

eFigure 1. Causal Diagram

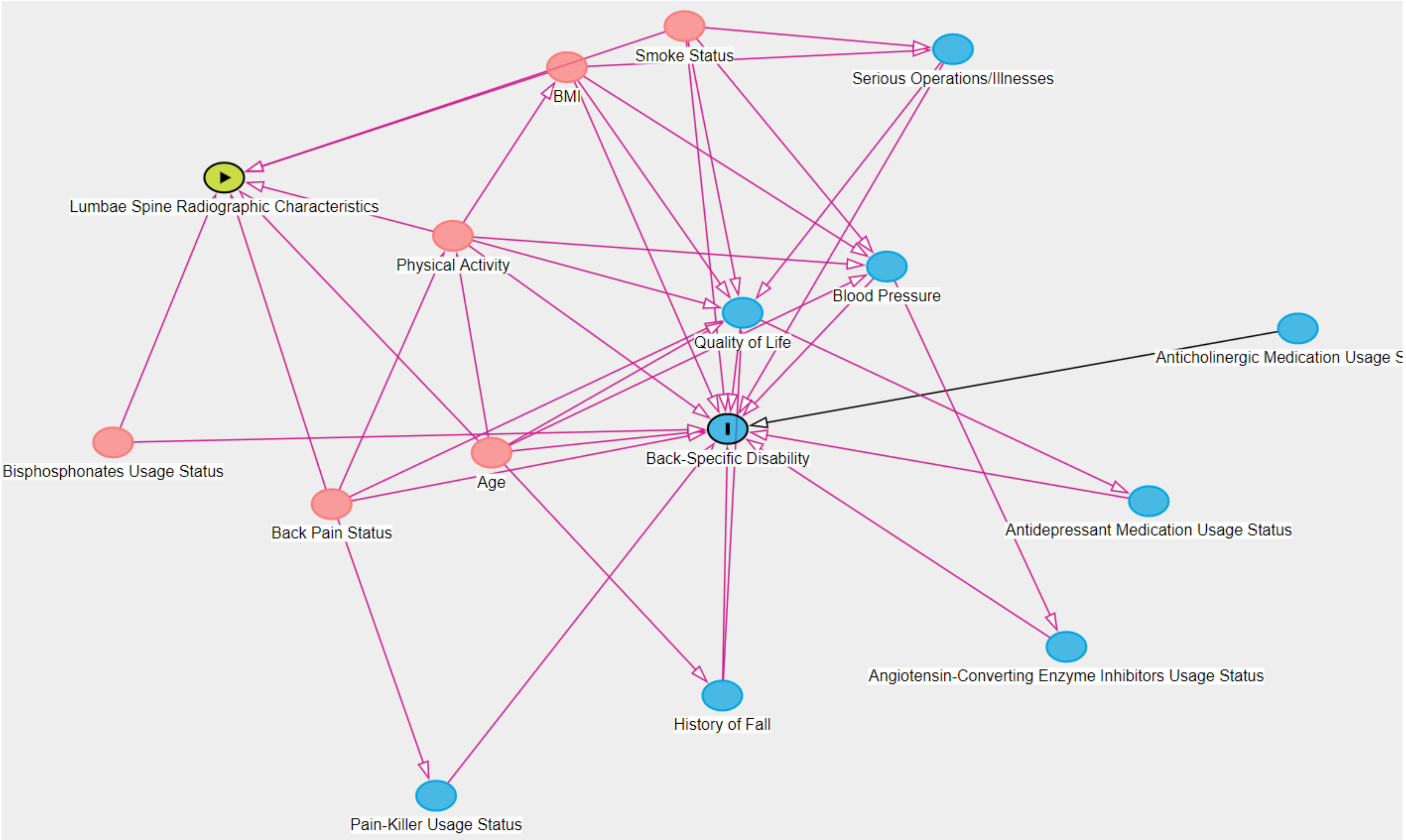

© 2021 Chen L et al. *JAMA Network Open.*

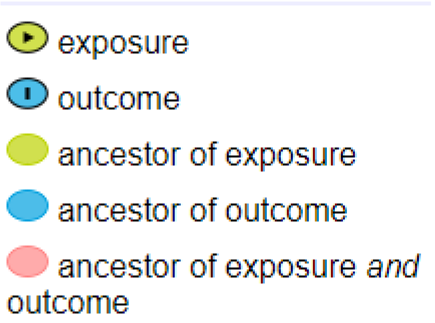

DAGitty is a browser-based environment for creating, editing, and analyzing causal diagrams (also known as directed acyclic graphs or causal Bayesian networks). The focus is on the use of causal diagrams for minimizing bias in empirical studies in epidemiology and other disciplines. We used two steps to choose the final covariates included in the model. At first, we pre-specified a lot of potential confounders based on clinical knowledge. The second step included searching previous literature for well accepted confounders and then drawing the causal diagram to find the minimal sufficient adjustment sets based on the established evidence.

Initially selected covariates: age, BMI, smoke status, back pain status, bisphosphonates usage status, physical activity, blood pressure, serious operations/illnesses, quality of life, history of fall, pain-killer usage status, anticholinergic medication usage status, antidepressant medication usage status, and angiotensin-converting enzyme inhibitors usage status.

Minimal sufficient adjustment sets: age (as a continuous variable), BMI (as a continuous variable), smoking status (never, current, and ex-smoker), back pain status (yes or no), bisphosphonates use (yes or no), and physical activity (as three domains: walking, job and sport; Walking [per week]: <0.5 miles, 0.5-5 miles, 5-10 miles, and 10+ miles. Job: sedentary, sedentary + occasional exercise, 0.5 sedentary + 0.5 active [or active housework, e.g., daily dust/hoover]), and predominantly manual, active all day. Sport: none, 1 hour per week golf, bowls, badminton, cycling or swimming, 2 hours previous or 1 hour keep-fit, aerobics, squash, and 2 hours + keep-fit, aerobics, squash).

© 2021 Chen L et al. *JAMA Network Open.*

eFigure 2. Histogram of Outcome Distribution

A. Cross-sectional part.

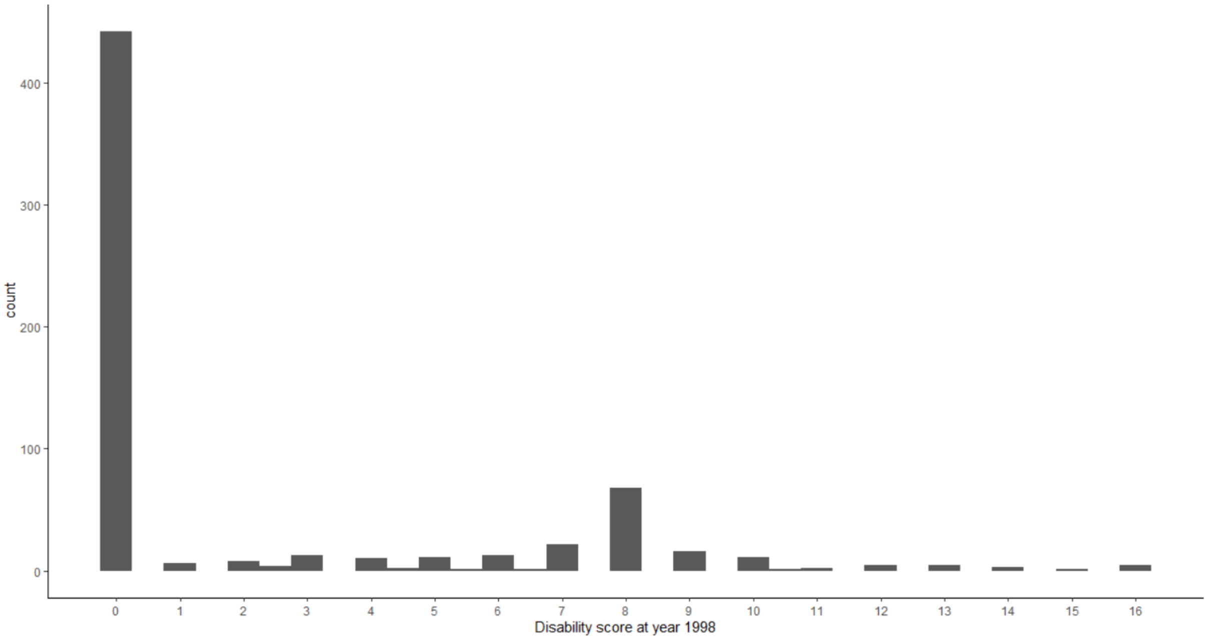

B. Longitudinal part.

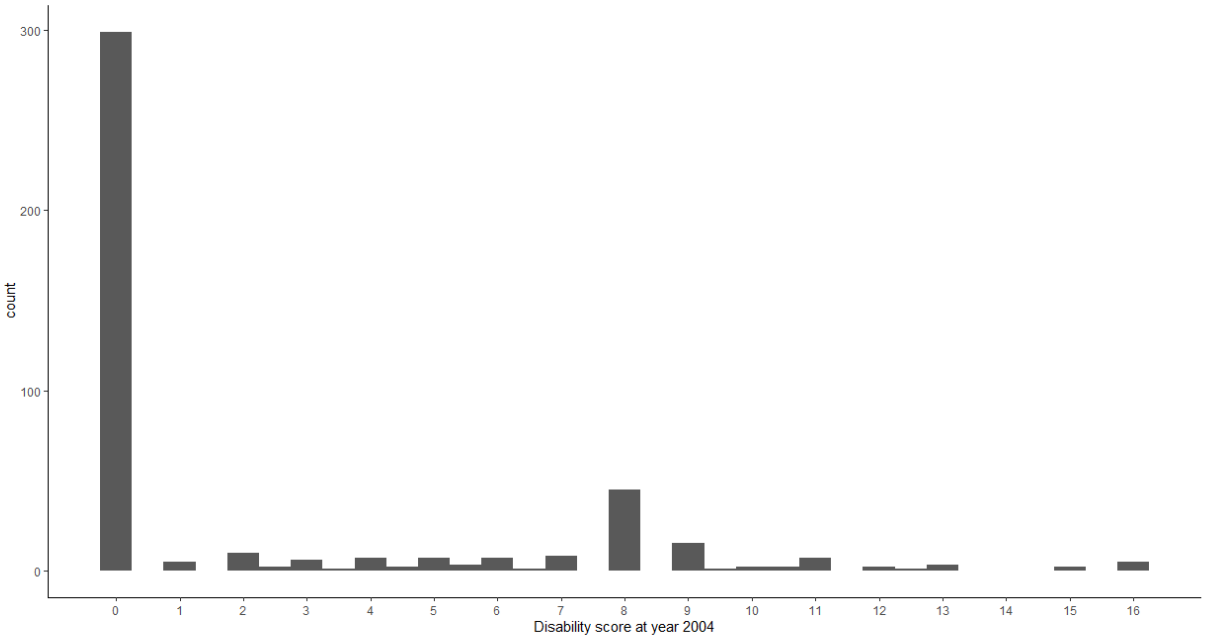

© 2021 Chen L et al. *JAMA Network Open.*

eFigure 3. Missing Data Pattern

A. Cross-sectional part.

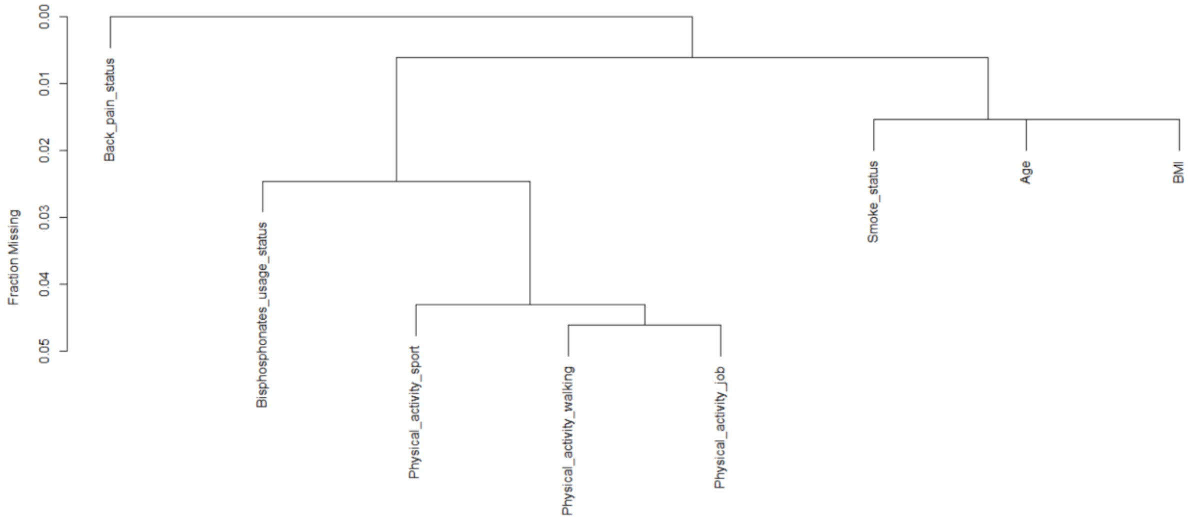

B. Longitudinal part.

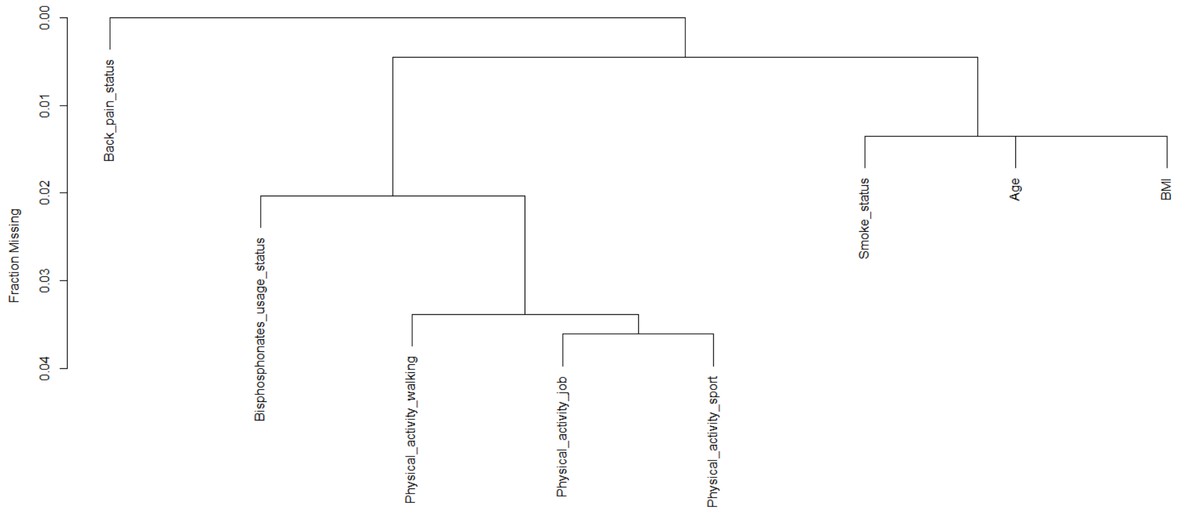

© 2021 Chen L et al. *JAMA Network Open.*

eAppendix 1. Methods for Exploratory and Sensitivity Analyses

Exploratory analyses

We examined whether the association between lumbar spine radiographic changes and the severity of back pain-related disability differed by age, BMI or smoking status through testing of multiplicative interactions using WALD statistics.

Sensitivity analyses

1) To assess the validity of our cut-off points for defining lumbar spine radiographic changes, we changed the cut-off points from 'no change vs any change' to 'no or mild change vs moderate-to-severe change'.

2) We changed the composite score by using the total original score, which is a sum score from the original grade score at each segment. For example, the K/L grade based on total original score ranged from 0-16 (at each segment 0-4).

3) We built a new composite score based on the disease severity: for disc space narrowing and osteophyte based score, grade 0 vs grade 2 and 3; for K/L grade based score, grade 0 and 1 vs grade 3 and 4.

4) Considering the potential heterogeneity of the population, we restricted our analyses to women with back pain.

5) Considering the potential model misspecification issue, we changed it to linear model and ordinal logistic regression with cloglog link function.

6) To explore the potential influence from unmeasured confounding, E-value was calculated.

7) As one reviewer suggested, we additionally adjusted pain medication and depression considering they might affect the results as potential strong prognostic factors.

© 2021 Chen L et al. *JAMA Network Open.*

eAppendix 2. Details of Statistical Methods

Ordinal logistic regression: Liu et al. indicated that ordinal regression models are robust for continuous outcomes, especially when the distributions of continuous responses are skewed (Stat Med. 2017 Nov 30;36(27):4316-4335). Our continuous outcomes were skewed so that we chose ordinal logistic regression.

Stepped modelling framework: If all covariates were measured at the same time, two models were sufficient: step 1: unadjusted analyses; step 2: analyses adjusted for age, BMI, back pain status, physical activity, bisphosphonates usage status, and smoking status (additionally adjusted for Year 9 back pain-related disability for the longitudinal analysis). The reason for the inclusion of Year 9 back pain-related disability for the longitudinal analysis is that it is a strong prognostic factor for Year 15 back pain-related disability. As VanderWeele et al. indicated, it is often important to control, whenever possible, for the outcome at or prior to the time of the baseline exposure assessment so that confounding control assumption is as plausible as possible (*Statistical Science* 35.3 (2020): 437-466.). In our study, among all selected covariates, physical activity was measured at Year 6 and others were measured at Year 9 (baseline at our study). Considering physical activity could change between Year 6 and Year 9, we built three models: step 1: unadjusted analyses; step 2: analyses adjusted for age, BMI, back pain status, bisphosphonates usage status, and smoking status (additionally adjusted for Year 9 back pain-related disability for the longitudinal analysis); step 3: analyses further adjusted for physical activity.

Exposure modelling: For each type of radiographic changes, our exposure has five values: 0, 1, 2, 3, and 4. The value equals the number of lumbar spine segments affected by radiographic

© 2021 Chen L et al. *JAMA Network Open.*

changes. We could consider the exposure as the unordered categorical variable. In this case, we set 0 as the reference level and obtained the estimate by comparing other values with 0. We could also consider 0-4 as the continuous variable. As a continuous variable, we tested the linear trend by modelling the exposure as continuous variable and reported the P-value (whether the regression coefficient of the exposure variable equalled zero); we also tested the non-linear trend by adding the quartic term to the previous model and reported the P-value (whether the added quartic term could improve performance of the previous model through analysis of variance).

Multiple imputation: It is a general approach to handle missing data in epidemiological and clinical research (BMJ 2009;338:b2393). It includes two steps: step 1: to create multiple copies of the dataset with the missing data replaced by imputed values; step 2: to fit the model to each of the imputed datasets and then calculate the final estimate by combing the estimate from each dataset using Rubin's rule (Rubin, Donald B. *Multiple imputation for nonresponse in surveys*. Vol. 81. John Wiley & Sons, 2004.).

© 2021 Chen L et al. *JAMA Network Open.*

eTable 1. Distribution of Lumbar Spine Radiographic Changes at Each Lumbar Spine Segment

	Cross-section (n=650)	Longitudinal (n=443)
**Kellgren-Lawrence grade**		
L1-L2		
grade 0	229	160
grade 1	199	140
grade 2	94	57
grade 3	96	68
grade 4	32	18
L2-L3		
grade 0	160	121
grade 1	183	126
grade 2	126	75
grade 3	137	92
grade 4	44	
L3-L4		
grade 0	102	72
grade 1	202	143
grade 2	146	93
grade 3	171	119
grade 4	29	16
L4-L5		
grade 0	135	89
grade 1	230	167
grade 2	88	61
grade 3	116	75
grade 4	81	51
**Disc space narrowing**		
L1-L2		
grade 0	311	219
grade 1	295	196
grade 2	29	23
grade 3	15	5
L2-L3		
grade 0	300	216
grade 1	296	197
grade 2	40	22
grade 3	14	8
L3-L4		
grade 0	276	198
grade 1	330	222
grade 2	31	15
grade 3	13	8
L4-L5		
grade 0	259	191
grade 1	280	182

© 2021 Chen L et al. *JAMA Network Open.*

grade 2	67	38
grade 3	44	32
**Osteophytes**		
L1-L2		
grade 0	551	383
grade 1	92	58
grade 2	7	2
grade 3	0	0
L2-L3		
grade 0	509	368
grade 1	129	71
grade 2	10	4
grade 3	2	0
L3-L4		
grade 0	468	335
grade 1	161	98
grade 2	18	10
grade 3	3	0
L4-L5		
grade 0	430	306
grade 1	191	118
grade 2	27	18
grade 3	2	1

© 2021 Chen L et al. *JAMA Network Open.*

eTable 2. Redundancy Analysis of Exposures

R-squared with which each variable can be predicted from all other variables.

R-squared cut-off: 0.75.

	R-squared
**Cross-sectional**	
L1-L2	
Osteophytes	0.157
Disc space narrowing	0.662
Kellgren-Lawrence grade	0.644
L2-L3	
Osteophytes	0.235
Disc space narrowing	0.649
Kellgren-Lawrence grade	0.633
L3-L4	
Osteophytes	0.198
Disc space narrowing	0.659
Kellgren-Lawrence grade	0.647
L4-L5	
Osteophytes	0.165
Disc space narrowing	0.721
Kellgren-Lawrence grade	0.717
**Longitudinal**	
L1-L2	
Osteophytes	0.020
Disc space narrowing	0.574
Kellgren-Lawrence grade	0.574
L2-L3	
Osteophytes	0.195
Disc space narrowing	0.616
Kellgren-Lawrence grade	0.603
L3-L4	
Osteophytes	0.147
Disc space narrowing	0.657
Kellgren-Lawrence grade	0.657
L4-L5	
Osteophytes	0.149
Disc space narrowing	0.725
Kellgren-Lawrence grade	0.727

© 2021 Chen L et al. *JAMA Network Open.*

eTable 3. Interaction with Age, BMI, or Smoking Status

	Cross-sectional (P-value)	Longitudinal (P-value)
Kellgren-Lawrence grade based score		
Age	0.84	0.61
BMI	0.62	0.23
Smoke status	0.18	0.66
Osteophytes grade based score		
Age	0.61	0.96
BMI	0.45	0.50
Smoke status	0.63	0.62
Disc space narrowing grade based score		
Age	0.28	0.65
BMI	0.88	0.14
Smoke status	0.41	0.20

© 2021 Chen L et al. *JAMA Network Open.*

eAppendix 3. Sensitivity Analyses Results: Change on the Cut-Off Points of Exposures (corresponding to the first sensitivity analysis; the cut-off points changed from 'no change vs any change' to 'no or mild change vs moderate-to-severe change').

eTable 4. Kellgren-Lawrence Grade–Based Score

**Variables**	**K/L grade based score**					**P for trend**	
	**0 segment**	**1 segment**	**2 segments**	**3 segments**	**4 segments**	**Linear model**	**Non-linear model**
**Cross-sectional (Year 9, n=650)**							
Number of women (%)	296 (45.5)	153 (23.5)	99 (15.2)	53 (8.2)	49 (7.5)		
Odds ratio (95% confidence interval)							
Unadjusted	1 (reference)	1.05 (0.70, 1.58)	1.24 (0.78, 1.98)	1.09 (0.60, 1.98)	1.02 (0.53, 1.93)	0.66	0.75
Multivariable adjusted[a]	1 (reference)	1.07 (0.70, 1.64)	1.31 (0.80, 2.13)	1.15 (0.61, 2.16)	1.32 (0.67, 2.62)	0.29	0.78
Further adjusted for physical activity	1 (reference)	1.06 (0.69, 1.63)	1.25 (0.76, 2.04)	1.12 (0.60, 2.12)	1.32 (0.66, 2.64)	0.34	0.89
**Longitudinal (Year 15, n=443)**							
Number of women (%)	200 (45.1)	110 (24.8)	68 (15.3)	38 (8.6)	27 (6.1)		
Odds ratio (95% confidence interval)							
Unadjusted	1 (reference)	1.16 (0.71, 1.88)	1.14 (0.64, 2.03)	1.34 (0.68, 2.66)	1.39 (0.62, 3.14)	0.29	0.57
Multivariable adjusted[b]	1 (reference)	1.10 (0.64, 1.88)	1.05 (0.54, 2.02)	1.38 (0.65, 2.95)	1.12 (0.45, 2.77)	0.56	0.87
Further adjusted for physical activity	1 (reference)	1.08 (0.62, 1.86)	1.00 (0.51, 1.97)	1.46 (0.66, 3.19)	1.15 (0.46, 2.90)	0.53	0.98

[a] Adjusted for age, BMI, smoke status, back pain status and bisphosphonates usage status.

[b] Adjusted for age, BMI, smoke status, back pain status, bisphosphonates usage status and Year 9 back pain-related disability.

© 2021 Chen L et al. *JAMA Network Open.*

eTable 5. Osteophytes Grade–Based Score

Variables	Osteophytes grade based score					P for trend	
	**0 segment**	**1 segment**	**2 segments**	**3 segments**	**4 segments**	**Linear model**	**Non-linear model**
**Cross-sectional (Year 9, n=650)**							
Number of women (%)	596 (91.7)	45 (6.9)	4 (0.6)	4 (0.6)	1 (0.2)		
Odds ratio (95% confidence interval)							
Unadjusted	1 (reference)	0.89 (0.47, 1.69)	0.56 (0.06, 5.02)	N/A	N/A	0.16	0.45
Multivariable adjusted[a]	1 (reference)	0.98 (0.51, 1.89)	0.53 (0.06, 4.89)	N/A	N/A	0.31	0.33
Further adjusted for physical activity	1 (reference)	1.00 (0.52, 1.97)	0.62 (0.07, 5.99)	N/A	N/A	0.35	0.33
**Longitudinal (Year 15, n=443)**							
Number of women (%)	411 (92.8)	30 (6.8)	1 (0.2)	1 (0.2)	0 (0.0)		
Odds ratio (95% confidence interval)							
Unadjusted	1 (reference)	0.55 (0.23, 1.28)	N/A	N/A	N/A	0.10	0.37
Multivariable adjusted[b]	1 (reference)	0.62 (0.24, 1.56)	N/A	N/A	N/A	0.18	0.85
Further adjusted for physical activity	1 (reference)	0.64 (0.25, 1.62)	N/A	N/A	N/A	0.18	0.88

[a] Adjusted for age, BMI, smoke status, back pain status and bisphosphonates usage status.

[b] Adjusted for age, BMI, smoke status, back pain status, bisphosphonates usage status and Year 9 back pain-related disability.

© 2021 Chen L et al. *JAMA Network Open.*

eTable 6. Disc Space Narrowing Grade–Based Score

**Variables**	**Disc space narrowing grade based score**					**P for trend**	
	**0 segment**	**1 segment**	**2 segments**	**3 segments**	**4 segments**	**Linear model**	**Non-linear model**
**Cross-sectional (Year 9, n=650)**							
Number of women (%)	480 (73.8)	114 (17.5)	37 (5.7)	11 (1.7)	8 (1.2)		
Odds ratio (95% confidence interval)							
Unadjusted	1 (reference)	1.39 (0.92, 2.11)	1.01 (0.51, 2.01)	0.75 (0.20, 2.77)	0.33 (0.04, 2.72)	0.87	0.18
Multivariable adjusted[a]	1 (reference)	1.44 (0.93, 2.22)	1.01 (0.49, 2.06)	0.88 (0.23, 3.42)	0.46 (0.05, 3.81)	0.88	0.11
Further adjusted for physical activity	1 (reference)	1.45 (0.93, 2.25)	1.00 (0.48, 2.06)	0.95 (0.24, 3.76)	0.46 (0.05, 3.94)	0.84	0.12
**Longitudinal (Year 15, n=443)**							
Number of women (%)	332 (74.9)	82 (18.5)	21 (4.7)	5 (1.1)	3 (0.7)		
Odds ratio (95% confidence interval)							
Unadjusted	1 (reference)	1.35 (0.83, 2.22)	1.75 (0.77, 3.97)	0.45 (0.05, 3.88)	N/A	0.64	0.13
Multivariable adjusted[b]	1 (reference)	1.28 (0.73, 2.23)	1.63 (0.67, 3.98)	0.61 (0.06, 6.65)	N/A	0.93	0.06
Further adjusted for physical activity	1 (reference)	1.41 (0.80, 2.49)	1.68 (0.68, 4.12)	0.67 (0.06, 7.59)	N/A	0.72	0.05

[a] Adjusted for age, BMI, smoke status, back pain status and bisphosphonates usage status.

[b] Adjusted for age, BMI, smoke status, back pain status, bisphosphonates usage status and Year 9 back pain-related disability.

© 2021 Chen L et al. *JAMA Network Open.*

eAppendix 4. Sensitivity Analyses Results: Total Original Score of Exposures (corresponding to the second sensitivity analysis; the total original score which is a sum score from the original grade score at each segment).

eTable 7. Kellgren-Lawrence Grade

	Linear model (effect estimate with its 95% confidence interval)	Non-linear model (p-value)
**Cross-sectional (Year 9, n=650)**		
Unadjusted	-0.12 (-0.34, 0.11)	0.26
Multivariable adjusted[a]	-0.07 (-0.31, 0.17)	0.19
Further adjusted for physical activity	-0.09 (-0.33, 0.16)	0.15
**Longitudinal (Year 15, n=443)**		
Unadjusted	-0.04 (-0.32, 0.23)	0.11
Multivariable adjusted[b]	-0.10 (-0.41, 0.21)	0.25
Further adjusted for physical activity	-0.11 (-0.43, 0.20)	0.13

[a] Adjusted for age, BMI, smoke status, back pain status and bisphosphonates usage status.

[b] Adjusted for age, BMI, smoke status, back pain status, bisphosphonates usage status and Year 9 back pain-related disability.

© 2021 Chen L et al. *JAMA Network Open.*

eTable 8. Osteophytes Grade

	Linear model	Non-linear model
**Cross-sectional (Year 9, n=650)**		
Unadjusted	-0.12 (-0.26, 0.01)	1.00
Multivariable adjusted[a]	-0.10 (-0.25, 0.05)	0.70
Further adjusted for physical activity	-0.10 (-0.24, 0.05)	0.71
**Longitudinal (Year 15, n=443)**		
Unadjusted	-0.25 (-0.44, -0.05)	0.79
Multivariable adjusted[b]	-0.26 (-0.48, -0.05)	0.99
Further adjusted for physical activity	-0.26 (-0.48, -0.04)	0.90

[a] Adjusted for age, BMI, smoke status, back pain status and bisphosphonates usage status.

[b] Adjusted for age, BMI, smoke status, back pain status, bisphosphonates usage status and Year 9 back pain-related disability.

© 2021 Chen L et al. *JAMA Network Open.*

eTable 9. Disc Space Narrowing Grade

	Linear model	Non-linear model
**Cross-sectional (Year 9, n=650)**		
Unadjusted	-0.07 (-0.30, 0.16)	0.06
Multivariable adjusted[a]	-0.03 (-0.29, 0.22)	0.07
Further adjusted for physical activity	-0.03 (-0.29, 0.22)	0.09
**Longitudinal (Year 15, n=443)**		
Unadjusted	0.16 (-0.13, 0.44)	0.98
Multivariable adjusted[b]	0.20 (-0.13, 0.52)	0.94
Further adjusted for physical activity	0.25 (-0.08, 0.59)	1.00

[a] Adjusted for age, BMI, smoke status, back pain status and bisphosphonates usage status.

[b] Adjusted for age, BMI, smoke status, back pain status, bisphosphonates usage status and Year 9 back pain-related disability.

© 2021 Chen L et al. *JAMA Network Open.*

eAppendix 5. Sensitivity Analyses Results: Disease Severity (corresponding to the third sensitivity analysis; a new composite score based on the disease severity).

eTable 10. Kellgren-Lawrence Grade–Based Score

**Variables**	**K/L grade based score**					**P for trend**	
	**0 segment**	**1 segment**	**2 segments**	**3 segments**	**4 segments**	**Linear model**	**Non-linear model**
**Cross-sectional (Year 9, n=365)**							
Number of women (%)	154 (42.2)	77 (21.1)	47 (12.9)	38 (10.4)	49 (13.4)		
Odds ratio (95% confidence interval)							
Unadjusted	1 (reference)	0.92 (0.52, 1.63)	1.27 (0.66, 2.44)	1.14 (0.56, 2.32)	0.92 (0.47, 1.82)	0.89	0.63
Multivariable adjusted[a]	1 (reference)	1.06 (0.59, 1.92)	1.33 (0.67, 2.65)	1.26 (0.58, 2.70)	1.34 (0.63, 2.84)	0.34	0.83
Further adjusted for physical activity	1 (reference)	1.03 (0.56, 1.88)	1.33 (0.66, 2.66)	1.31 (0.60, 2.89)	1.35 (0.63, 2.88)	0.31	0.87
**Longitudinal (Year 15, n=260)**							
Number of women (%)	112 (43.1)	60 (23.1)	34 (13.1)	27 (10.4)	27 (10.4)		
Odds ratio (95% confidence interval)							
Unadjusted	1 (reference)	1.31 (0.70, 2.48)	1.49 (0.70, 3.17)	1.15 (0.50, 2.67)	1.25 (0.53, 2.91)	0.51	0.42
Multivariable adjusted[b]	1 (reference)	1.32 (0.65, 2.67)	1.25 (0.52, 2.99)	1.37 (0.54, 3.50)	1.12 (0.42, 2.94)	0.61	0.47
Further adjusted for physical activity	1 (reference)	1.36 (0.66, 2.82)	1.18 (0.47, 2.92)	1.71 (0.63, 4.67)	1.31 (0.47, 3.65)	0.40	0.57

[a] Adjusted for age, BMI, smoke status, back pain status and bisphosphonates usage status.

[b] Adjusted for age, BMI, smoke status, back pain status, bisphosphonates usage status and Year 9 back pain-related disability.

© 2021 Chen L et al. *JAMA Network Open.*

eTable 11. Osteophytes Grade–Based Score

Variables	Osteophytes grade based score					P for trend	
	0 segment	1 segment	2 segments	3 segments	4 segments	Linear model	Non-linear model
**Cross-sectional (Year 9, n=283)**							
Number of women (%)	258 (91.2)	20 (7.1)	1 (0.4)	3 (1.1)	1 (0.4)		
Odds ratio (95% confidence interval)							
Unadjusted	1 (reference)	0.56 (0.20, 1.56)	2.29 (0.14, 37.06)	N/A	N/A	0.14	0.71
Multivariable adjusted[a]	1 (reference)	0.49 (0.17, 1.39)	1.02 (0.06, 17.96)	N/A	N/A	0.13	0.94
Further adjusted for physical activity	1 (reference)	0.50 (0.17, 1.46)	0.80 (0.04, 16.41)	N/A	N/A	0.11	0.88
**Longitudinal (Year 15, n=206)**							
Number of women (%)	192 (93.2)	12 (5.8)	1 (0.5)	1 (0.5)	0 (0.0)		
Odds ratio (95% confidence interval)							
Unadjusted	1 (reference)	0.56 (0.15, 2.09)	N/A	N/A	N/A	0.20	0.89
Multivariable adjusted[b]	1 (reference)	0.87 (0.19, 4.00)	N/A	N/A	N/A	0.49	0.88
Further adjusted for physical activity	1 (reference)	1.41 (0.24, 8.33)	N/A	N/A	N/A	0.71	0.87

[a] Adjusted for age, BMI, smoke status, back pain status and bisphosphonates usage status.

[b] Adjusted for age, BMI, smoke status, back pain status, bisphosphonates usage status and Year 9 back pain-related disability.

© 2021 Chen L et al. *JAMA Network Open.*

eTable 12. Disc Space Narrowing Grade–Based Score

Variables	Disc space narrowing grade based score					P for trend	
	**0 segment**	**1 segment**	**2 segments**	**3 segments**	**4 segments**	**Linear model**	**Non-linear model**
**Cross-sectional (Year 9, n=126)**							
Number of women (%)	100 (79.4)	11 (8.7)	5 (4.0)	2 (1.6)	8 (6.3)		
Odds ratio (95% confidence interval)							
Unadjusted	1 (reference)	1.69 (0.46, 6.24)	1.45 (0.26, 8.15)	N/A	0.38 (0.04, 3.20)	0.44	0.25
Multivariable adjusted[a]	1 (reference)	1.33 (0.32, 5.50)	2.03 (0.30, 13.90)	N/A	0.53 (0.06, 5.14)	N/A	N/A
Further adjusted for physical activity	1 (reference)	1.20 (0.26, 5.56)	3.25 (0.37, 28.69)	N/A	0.59 (0.05, 6.89)	N/A	N/A
**Longitudinal (Year 15, n=87)**							
Number of women (%)	70 (80.5)	9 (10.3)	4 (4.6)	1 (1.1)	3 (3.4)		
Odds ratio (95% confidence interval)							
Unadjusted	1 (reference)	1.15 (0.27, 4.99)	0.53 (0.06, 4.98)	1.95 (0.12, 32.49)	N/A	0.39	0.47
Multivariable adjusted[b]	1 (reference)	1.76 (0.17, 18.44)	0.54 (0.03, 9.08)	0.02 (0.0001, 3.28)	N/A	0.33	0.19
Further adjusted for physical activity	1 (reference)	0.69 (0.09, 5.38)	0.06 (0.002, 1.38)	8.07 (0.12, 54.29)	N/A	0.16	0.89

[a] Adjusted for age, BMI, smoke status, back pain status and bisphosphonates usage status.

[b] Adjusted for age, BMI, smoke status, back pain status, bisphosphonates usage status and Year 9 back pain-related disability.

© 2021 Chen L et al. *JAMA Network Open.*

eAppendix 6. Sensitivity Analyses Results: Restricting to Women With Back Pain (corresponding to the fourth sensitivity analysis; the potential heterogeneity of the population).

eTable 13. Kellgren-Lawrence Grade–Based Score

**Variables**	**K/L grade based score**					**P for trend**	
	**0 segment**	**1 segment**	**2 segments**	**3 segments**	**4 segments**	**Linear model**	**Non-linear model**
**Cross-sectional (Year 9, n=210)**							
Number of women (%)	51 (24.3)	46 (21.9)	39 (18.6)	42 (20.0)	32 (15.2)		
Odds ratio (95% confidence interval)							
Unadjusted	1 (reference)	0.60 (0.26, 1.38)	0.73 (0.31, 1.71)	0.66 (0.29, 1.54)	0.53 (0.21, 1.33)	0.23	0.74
Multivariable adjusted[a]	1 (reference)	0.54 (0.15, 1.95)	0.29 (0.07, 1.17)	0.77 (0.21, 2.86)	0.21 (0.04, 1.13)	0.14	0.85
Further adjusted for physical activity	1 (reference)	0.74 (0.18, 3.10)	0.23 (0.05, 1.07)	0.76 (0.17, 3.37)	0.24 (0.04, 1.44)	0.14	0.66
**Longitudinal (Year 15, n=145)**							
Number of women (%)	40 (27.6)	32 (22.1)	24 (16.6)	26 (17.9)	23 (15.9)		
Odds ratio (95% confidence interval)							
Unadjusted	1 (reference)	1.05 (0.40, 2.74)	1.16 (0.42, 3.26)	0.43 (0.14, 1.38)	1.43 (0.52, 3.94)	0.93	0.49
Multivariable adjusted[b]	1 (reference)	0.75 (0.24, 2.36)	1.93 (0.60, 6.15)	0.56 (0.15, 2.12)	1.81 (0.54, 6.10)	0.43	0.62
Further adjusted for physical activity	1 (reference)	0.53 (0.15, 1.85)	1.57 (0.42, 5.78)	0.41 (0.09, 1.75)	1.92 (0.52, 7.06)	0.53	0.32

[a] Adjusted for age, BMI, smoke status and bisphosphonates usage status.

[b] Adjusted for age, BMI, smoke status, back pain status, bisphosphonates usage status and Year 9 back pain-related disability.

© 2021 Chen L et al. *JAMA Network Open.*

eTable 14. Osteophytes Grade–Based Score

Variables	Osteophytes grade based score					P for trend	
	**0 segment**	**1 segment**	**2 segments**	**3 segments**	**4 segments**	**Linear model**	**Non-linear model**
**Cross-sectional (Year 9, n=210)**							
Number of women (%)	81 (38.6)	76 (36.2)	32 (15.2)	15 (7.1)	6 (2.9)		
Odds ratio (95% confidence interval)							
Unadjusted	1 (reference)	0.70 (0.36, 1.34)	0.88 (0.38, 2.02)	0.60 (0.18, 2.03)	0.35 (0.04, 3.14)	0.27	0.98
Multivariable adjusted[a]	1 (reference)	1.05 (0.04, 2.76)	1.65 (0.48, 5.62)	0.74 (0.11, 4.83)	N/A	0.79	0.24
Further adjusted for physical activity	1 (reference)	0.97 (0.35, 2.73)	1.49 (0.41, 5.41)	1.08 (0.16, 7.34)	N/A	0.88	0.34
**Longitudinal (Year 15, n=145)**							
Number of women (%)	56 (38.6)	59 (40.7)	20 (13.8)	8 (5.5)	2 (1.4)		
Odds ratio (95% confidence interval)							
Unadjusted	1 (reference)	0.77 (0.37, 1.63)	0.64 (0.22, 1.87)	0.55 (0.10, 2.88)	N/A	0.18	0.74
Multivariable adjusted[b]	1 (reference)	0.91 (0.39, 2.16)	0.68 (0.20, 2.25)	0.67 (0.10, 4.68)	N/A	0.39	0.66
Further adjusted for physical activity	1 (reference)	0.90 (0.36, 2.25)	0.39 (0.10, 1.51)	0.58 (0.08, 4.29)	N/A	0.18	0.65

[a] Adjusted for age, BMI, smoke status and bisphosphonates usage status.

[b] Adjusted for age, BMI, smoke status, back pain status, bisphosphonates usage status and Year 9 back pain-related disability.

© 2021 Chen L et al. *JAMA Network Open.*

eTable 15. Disc Space Narrowing Grade–Based Score

Variables	Disc space narrowing grade based score					P for trend	
	**0 segment**	**1 segment**	**2 segments**	**3 segments**	**4 segments**	**Linear model**	**Non-linear model**
**Cross-sectional (Year 9, n=210)**							
Number of women (%)	25 (11.9)	27 (12.9)	45 (21.4)	41 (19.5)	72 (34.3)		
Odds ratio (95% confidence interval)							
Unadjusted	1 (reference)	0.94 (0.34, 2.64)	0.40 (0.15, 1.10)	0.55 (0.20, 1.47)	0.39 (0.15, 0.99)	0.03	0.58
Multivariable adjusted[a]	1 (reference)	0.81 (0.18, 3.55)	0.29 (0.06, 1.34)	0.55 (0.10, 2.93)	0.32 (0.07, 1.35)	0.10	0.53
Further adjusted for physical activity	1 (reference)	0.43 (0.08, 2.28)	0.23 (0.04, 1.23)	0.39 (0.06, 2.38)	0.26 (0.05, 1.30)	0.19	0.32
**Longitudinal (Year 15, n=145)**							
Number of women (%)	14 (9.7)	27 (18.6)	31 (21.4)	28 (19.3)	45 (31.0)		
Odds ratio (95% confidence interval)							
Unadjusted	1 (reference)	0.50 (0.15, 1.68)	0.22 (0.06, 0.79)	0.25 (0.07, 0.91)	0.83 (0.28, 2.45)	0.87	0.002
Multivariable adjusted[b]	1 (reference)	0.60 (0.15, 2.39)	0.43 (0.09, 2.00)	0.55 (0.12, 2.45)	1.47 (0.38, 5.75)	0.23	0.04
Further adjusted for physical activity	1 (reference)	0.55 (0.11, 2.66)	0.29 (0.05, 1.61)	0.53 (0.10, 2.80)	1.28 (0.27, 6.07)	0.25	0.03

[a] Adjusted for age, BMI, smoke status and bisphosphonates usage status.

[b] Adjusted for age, BMI, smoke status, back pain status, bisphosphonates usage status and Year 9 back pain-related disability.

© 2021 Chen L et al. *JAMA Network Open.*

eAppendix 7. Sensitivity Analyses Results: Changing the Model to cloglog Link Function (corresponding to the fifth sensitivity analysis; the potential model misspecification).

eTable 16. Kellgren-Lawrence Grade–Based Score

**Variables**	**K/L grade based score**					**P for trend**	
	**0 segment**	**1 segment**	**2 segments**	**3 segments**	**4 segments**	**Linear model**	**Non-linear model**
**Cross-sectional (Year 9, n=650)**							
Number of women (%)	154 (23.7)	142 (21.8)	140 (21.5)	118 (18.2)	96 (14.8)		
Odds ratio (95% confidence interval)							
Unadjusted	1 (reference)	1.16 (0.79, 1.70)	0.86 (0.57, 1.30)	0.89 (0.58, 1.37)	0.85 (0.53, 1.34)	0.24	0.82
Multivariable adjusted[a]	1 (reference)	1.20 (0.81, 1.77)	0.86 (0.57, 1.30)	0.93 (0.60, 1.44)	0.91 (0.56, 1.46)	0.36	0.93
Further adjusted for physical activity	1 (reference)	1.18 (0.80, 1.75)	0.84 (0.55, 1.28)	0.89 (0.57, 1.39)	0.89 (0.55, 1.44)	0.30	0.99
**Longitudinal (Year 15, n=443)**							
Number of women (%)	112 (25.3)	100 (22.6)	97 (21.9)	76 (17.2)	58 (13.1)		
Odds ratio (95% confidence interval)							
Unadjusted	1 (reference)	1.18 (0.75, 1.87)	0.95 (0.59, 1.55)	0.81 (0.48, 1.40)	1.07 (0.62, 1.84)	0.63	0.90
Multivariable adjusted[b]	1 (reference)	0.89 (0.54, 1.46)	0.91 (0.55, 1.53)	0.74 (0.42, 1.31)	0.84 (0.47, 1.52)	0.28	0.60
Further adjusted for physical activity	1 (reference)	0.86 (0.52, 1.43)	0.89 (0.53, 1.49)	0.72 (0.40, 1.28)	0.83 (0.45, 1.50)	0.25	0.54

[a] Adjusted for age, BMI, smoke status, back pain status and bisphosphonates usage status.

[b] Adjusted for age, BMI, smoke status, back pain status, bisphosphonates usage status and Year 9 back pain-related disability.

© 2021 Chen L et al. *JAMA Network Open.*

eTable 17. Osteophytes Grade–Based Score

Variables	Osteophytes grade based score					P for trend	
	**0 segment**	**1 segment**	**2 segments**	**3 segments**	**4 segments**	**Linear model**	**Non-linear model**
**Cross-sectional (Year 9, n=650)**							
Number of women (%)	258 (39.7)	226 (34.8)	102 (15.7)	44 (6.8)	20 (3.1)		
Odds ratio (95% confidence interval)							
Unadjusted	1 (reference)	0.84 (0.62, 1.15)	0.84 (0.56, 1.26)	0.59 (0.31, 1.13)	0.83 (0.36, 1.90)	0.12	0.64
Multivariable adjusted[a]	1 (reference)	0.87 (0.63, 1.19)	0.83 (0.55, 1.27)	0.66 (0.34, 1.30)	0.99 (0.42, 2.32)	0.29	0.50
Further adjusted for physical activity	1 (reference)	0.86 (0.63, 1.19)	0.85 (0.56, 1.31)	0.68 (0.35, 1.34)	0.97 (0.41, 2.30)	0.32	0.55
**Longitudinal (Year 15, n=443)**							
Number of women (%)	192 (43.3)	157 (35.4)	67 (15.1)	19 (4.3)	8 (1.8)		
Odds ratio (95% confidence interval)							
Unadjusted	1 (reference)	0.77 (0.53, 1.11)	0.68 (0.41, 1.13)	0.49 (0.18, 1.34)	0.58 (0.14, 2.37)	0.04	0.71
Multivariable adjusted[b]	1 (reference)	0.91 (0.62, 1.33)	0.68 (0.40, 1.16)	0.52 (0.18, 1.45)	0.48 (0.11, 2.06)	0.03	0.97
Further adjusted for physical activity	1 (reference)	0.94 (0.63, 1.39)	0.63 (0.36, 1.10)	0.55 (0.19, 1.57)	0.54 (0.12, 2.33)	0.03	0.93

[a] Adjusted for age, BMI, smoke status, back pain status and bisphosphonates usage status.

[b] Adjusted for age, BMI, smoke status, back pain status, bisphosphonates usage status and Year 9 back pain-related disability.

© 2021 Chen L et al. *JAMA Network Open.*

eTable 18. Disc Space Narrowing Grade–Based Score

Variables	Disc space narrowing grade based score					P for trend	
	**0 segment**	**1 segment**	**2 segments**	**3 segments**	**4 segments**	**Linear model**	**Non-linear model**
**Cross-sectional (Year 9, n=650)**							
Number of women (%)	100 (15.4)	107 (16.5)	147 (22.6)	131 (20.2)	165 (25.4)		
Odds ratio (95% confidence interval)							
Unadjusted	1 (reference)	1.28 (0.79, 2.10)	1.26 (0.79, 1.99)	1.31 (0.82, 2.10)	0.99 (0.62, 1.58)	0.80	0.10
Multivariable adjusted[a]	1 (reference)	1.36 (0.83, 2.25)	1.49 (0.92, 2.39)	1.42 (0.88, 2.30)	1.10 (0.67, 1.81)	0.87	0.04
Further adjusted for physical activity	1 (reference)	1.33 (0.80, 2.21)	1.47 (0.91, 2.38)	1.40 (0.86, 2.27)	1.06 (0.65, 1.75)	0.98	0.03
**Longitudinal (Year 15, n=443)**							
Number of women (%)	70 (15.8)	84 (19.0)	102 (23.0)	88 (19.9)	99 (22.3)		
Odds ratio (95% confidence interval)							
Unadjusted	1 (reference)	0.90 (0.52, 1.57)	0.79 (0.46, 1.36)	1.01 (0.59, 1.73)	1.15 (0.69, 1.93)	0.42	0.21
Multivariable adjusted[b]	1 (reference)	0.86 (0.48, 1.54)	0.89 (0.50, 1.59)	1.08 (0.61, 1.90)	1.37 (0.78, 2.39)	0.13	0.23
Further adjusted for physical activity	1 (reference)	0.86 (0.46, 1.59)	0.84 (0.46, 1.54)	1.16 (0.64, 2.10)	1.51 (0.84, 2.73)	0.08	0.14

[a] Adjusted for age, BMI, smoke status, back pain status and bisphosphonates usage status.

[b] Adjusted for age, BMI, smoke status, back pain status, bisphosphonates usage status and Year 9 back pain-related disability.

© 2021 Chen L et al. *JAMA Network Open.*

eAppendix 8. Sensitivity Analyses Results: Change the Model to Linear Regression (corresponding to the fifth sensitivity analysis; the potential model misspecification).

eTable 19. Kellgren-Lawrence Grade–Based Score

**Variables**	**K/L grade based score**					**P for trend**	
	**0 segment**	**1 segment**	**2 segments**	**3 segments**	**4 segments**	**Linear model**	**Non-linear model**
**Cross-sectional (Year 9, n=650)**							
Number of women (%)	154 (23.7)	142 (21.8)	140 (21.5)	118 (18.2)	96 (14.8)		
Odds ratio (95% confidence interval)							
Unadjusted	1 (reference)	1.42 (0.59, 3.40)	0.86 (0.36, 2.06)	0.99 (0.40, 2.50)	0.65 (0.25, 1.74)	0.31	0.49
Multivariable adjusted[a]	1 (reference)	1.44 (0.61, 3.42)	0.88 (0.37, 2.09)	1.01 (0.40, 2.55)	0.73 (0.27, 1.99)	0.43	0.55
Further adjusted for physical activity	1 (reference)	1.40 (0.59, 3.36)	0.81 (0.34, 1.94)	0.96 (0.38, 2.42)	0.69 (0.25, 1.88)	0.35	0.61
**Longitudinal (Year 15, n=443)**							
Number of women (%)	112 (25.3)	100 (22.6)	97 (21.9)	76 (17.2)	58 (13.1)		
Odds ratio (95% confidence interval)							
Unadjusted	1 (reference)	1.41 (0.48, 4.09)	0.91 (0.31, 2.68)	0.56 (0.18, 1.77)	0.89 (0.25, 3.13)	0.38	0.93
Multivariable adjusted[b]	1 (reference)	0.93 (0.36, 2.44)	1.01 (0.38, 2.67)	0.60 (0.21, 1.74)	0.54 (0.17, 1.75)	0.14	0.89
Further adjusted for physical activity	1 (reference)	0.92 (0.35, 2.43)	1.00 (0.38, 2.68)	0.59 (0.20, 1.72)	0.54 (0.16, 1.74)	0.13	0.87

[a] Adjusted for age, BMI, smoke status, back pain status and bisphosphonates usage status.

[b] Adjusted for age, BMI, smoke status, back pain status, bisphosphonates usage status and Year 9 back pain-related disability.

© 2021 Chen L et al. *JAMA Network Open.*

eTable 20. Osteophytes Grade–Based Score

**Variables**	**Osteophytes grade based score**					**P for trend**	
	**0 segment**	**1 segment**	**2 segments**	**3 segments**	**4 segments**	**Linear model**	**Non-linear model**
**Cross-sectional (Year 9, n=650)**							
Number of women (%)	258 (39.7)	226 (34.8)	102 (15.7)	44 (6.8)	20 (3.1)		
Odds ratio (95% confidence interval)							
Unadjusted	1 (reference)	0.64 (0.32, 1.27)	0.64 (0.26, 1.54)	0.35 (0.26, 1.20)	0.68 (0.12, 3.90)	0.11	0.47
Multivariable adjusted[a]	1 (reference)	0.68 (0.35, 1.34)	0.61 (0.25, 1.48)	0.42 (0.12, 1.46)	0.92 (0.16, 5.43)	0.22	0.36
Further adjusted for physical activity	1 (reference)	0.67 (0.34, 1.32)	0.63 (0.26, 1.55)	0.43 (0.12, 1.52)	0.84 (0.14, 5.01)	0.21	0.41
**Longitudinal (Year 15, n=443)**							
Number of women (%)	192 (43.3)	157 (35.4)	67 (15.1)	19 (4.3)	8 (1.8)		
Odds ratio (95% confidence interval)							
Unadjusted	1 (reference)	0.46 (0.20, 1.05)	0.28 (0.09, 0.83)	0.20 (0.03, 1.25)	0.18 (0.01, 2.96)	0.004	0.47
Multivariable adjusted[b]	1 (reference)	0.58 (0.28, 1.24)	0.27 (0.10, 0.74)	0.32 (0.06, 1.74)	0.12 (0.01, 1.41)	0.002	0.61
Further adjusted for physical activity	1 (reference)	0.59 (0.27, 1.26)	0.27 (0.10, 0.76)	0.32 (0.06, 1.76)	0.13 (0.01, 1.63)	0.002	0.57

[a] Adjusted for age, BMI, smoke status, back pain status and bisphosphonates usage status.

[b] Adjusted for age, BMI, smoke status, back pain status, bisphosphonates usage status and Year 9 back pain-related disability.

© 2021 Chen L et al. *JAMA Network Open.*

eTable 21. Disc Space Narrowing Grade–Based Score

Variables	Disc space narrowing grade based score					P for trend	
	**0 segment**	**1 segment**	**2 segments**	**3 segments**	**4 segments**	**Linear model**	**Non-linear model**
**Cross-sectional (Year 9, n=650)**							
Number of women (%)	100 (15.4)	107 (16.5)	147 (22.6)	131 (20.2)	165 (25.4)		
Odds ratio (95% confidence interval)							
Unadjusted	1 (reference)	1.59 (0.56, 4.52)	1.68 (0.63, 4.45)	1.43 (0.53, 3.90)	0.92 (0.35, 2.39)	0.62	0.11
Multivariable adjusted[a]	1 (reference)	1.70 (0.60, 4.82)	2.07 (0.78, 5.46)	1.52 (0.56, 4.12)	0.98 (0.36, 2.63)	0.69	0.05
Further adjusted for physical activity	1 (reference)	1.61 (0.56, 4.61)	2.00 (0.75, 5.34)	1.50 (0.55, 4.10)	0.94 (0.35, 2.56)	0.65	0.05
**Longitudinal (Year 15, n=443)**							
Number of women (%)	70 (15.8)	84 (19.0)	102 (23.0)	88 (19.9)	99 (22.3)		
Odds ratio (95% confidence interval)							
Unadjusted	1 (reference)	0.96 (0.27, 3.36)	0.75 (0.23, 2.51)	1.55 (0.45, 5.36)	1.83 (0.55, 6.15)	0.18	0.36
Multivariable adjusted[b]	1 (reference)	0.64 (0.20, 2.01)	0.67 (0.22, 2.03)	1.22 (0.39, 3.77)	1.35 (0.43, 4.31)	0.14	0.22
Further adjusted for physical activity	1 (reference)	0.60 (0.19, 1.95)	0.63 (0.20, 1.92)	1.19 (0.38, 3.72)	1.39 (0.43, 4.49)	0.14	0.18

[a] Adjusted for age, BMI, smoke status, back pain status and bisphosphonates usage status.

[b] Adjusted for age, BMI, smoke status, back pain status, bisphosphonates usage status and Year 9 back pain-related disability.

© 2021 Chen L et al. *JAMA Network Open.*

eTable 22. Additionally Adjusted for Pain Medication and Depression (corresponding to the seventh sensitivity analysis; potential strong prognostic factors).

	Cross-sectional (n=650)					
	0 segment (n=154)	1 segment (n=142)	2 segments (n=140)	3 segments (n=118)	4 segments (n=96)	Whole (n=650)
Pain Medication						
Yes	10 (6.5)	7 (4.9)	16 (11.4)	8 (6.8)	10 (10.4)	51 (7.8)
No	68 (44.2)	60 (42.3)	59 (42.1)	53 (44.9)	37 (38.5)	277 (42.6)
Missing	76 (49.4)	75 (52.8)	65 (46.4)	57 (48.3)	49 (51.1)	322 (49.5)
Depression						
Yes	0 (0.0)	1 (0.7)	0 (0.0)	1 (0.8)	1 (1.0)	3 (0.5)
No	154 (100.0)	141 (99.3)	140 (100.0)	117 (99.2)	95 (99.0)	647 (99.5)
	Longitudinal (n=443)					
	0 segment (n=112)	1 segment (n=100)	2 segments (n=97)	3 segments (n=76)	4 segments (n=58)	Whole (n=443)
Pain Medication						
Yes	10 (8.9)	6 (6.0)	9 (9.3)	4 (5.3)	6 (10.3)	35 (7.9)
No	42 (37.5)	42 (42.0)	48 (49.5)	35 (46.1)	24 (41.4)	191 (43.1)

© 2021 Chen L et al. *JAMA Network Open.*

Missing	60 (53.6)	52 (52.0)	40 (41.2)	37 (48.7)	28 (48.3)	217 (49.0)
Depression						
Yes	0 (0.0)	1 (1.0)	1 (1.0)	1 (1.3)	1 (1.7)	3 (0.7)
No	112 (100.0)	99 (99.0)	96 (99.0)	75 (98.7)	57 (98.3)	443 (99.3)

The columns except the first correspond to the number of segments of Lumbar Spine Radiographic Changes (Kellgren-Lawrence grade based). Data are present as number (percentage) of participants unless otherwise indicated.

Women reported current medication use in an open field question within the medical history questionnaire. Data on use of non-opioid and opioid analgesics, defined based on Anatomical Therapeutic Chemical codes M01 and N02, were extracted from this question from Year 9. The details are:

With specific name

With opioid involved

Dihydrocodeine; Dextromoramide; Tramadol; Codeine; Morphine; Paracetamol and dextropropoxyphene; Paracetamol and Codeine; Paracetamol and dihydrocodeine

Without opioid involved

Indomethacin; Ibuprofen; Diclofenac; Etodolac; Fenbufen; Flurbiprofen; Fenoprofen; Mefenamic acid; Naproxen; Piroxicam; Ketoprofen; Movelat; Glucosamine; Feverfew;

Paracetamol

Without specific name

NSAID; Anti-inflammatory; Analgesics; Painkillers

Depression was defined by text response. From Year 1 to Year 4, women were asked the question: Serious operations/illnesses: Other? From Year 8 to Year 9, women were asked the question: Any major illnesses or operations? If the participant reported depression in at least one year (Year 1 to 9, our baseline is Year 9), we defined the value of this covariate as yes.

© 2021 Chen L et al. *JAMA Network Open.*

eTable 23. Kellgren-Lawrence Grade–Based Score

Variables	K/L grade based score					P for trend	
	**0 segment**	**1 segment**	**2 segments**	**3 segments**	**4 segments**	**Linear model**	**Non-linear model**
**Cross-sectional (Year 9, n=650)**							
Number of women (%)	154 (23.7)	142 (21.8)	140 (21.5)	118 (18.2)	96 (14.8)		
Odds ratio (95% confidence interval)							
Additional adjusted[a]	1 (reference)	1.21 (0.61, 2.42)	0.85 (0.42, 1.75)	1.25 (0.60, 2.63)	0.87 (0.38, 2.02)	0.84	0.76
**Longitudinal (Year 15, n=443)**							
Number of women (%)	112 (25.3)	100 (22.6)	97 (21.9)	76 (17.2)	58 (13.1)		
Odds ratio (95% confidence interval)							
Additional adjusted[b]	1 (reference)	1.10 (0.59, 2.04)	0.92 (0.49, 1.74)	0.66 (0.33, 1.33)	0.78 (0.37, 1.62)	0.14	0.86

[a] Adjusted for age, BMI, smoke status, back pain status, bisphosphonates usage status, physical activity, pain medication and depression.

[b] Adjusted for age, BMI, smoke status, back pain status, bisphosphonates usage status, physical activity, pain medication, depression and Year 9 back pain-related disability.

© 2021 Chen L et al. *JAMA Network Open.*

eTable 24. Osteophytes Grade–Based Score

Variables	Osteophyte grade based score					P for trend	
	**0 segment**	**1 segment**	**2 segments**	**3 segments**	**4 segments**	**Linear model**	**Non-linear model**
**Cross-sectional (Year 9, n=650)**							
Number of women (%)	154 (23.7)	142 (21.8)	140 (21.5)	118 (18.2)	96 (14.8)		
Odds ratio (95% confidence interval)							
Additional adjusted[a]	1 (reference)	1.17 (0.67, 2.05)	1.25 (0.63, 2.48)	0.66 (0.22, 1.99)	2.46 (0.70, 8.56)	0.52	0.86
**Longitudinal (Year 15, n=443)**							
Number of women (%)	112 (25.3)	100 (22.6)	97 (21.9)	76 (17.2)	58 (13.1)		
Odds ratio (95% confidence interval)							
Additional adjusted[b]	1 (reference)	0.87 (0.44, 1.73)	0.54 (0.22, 1.32)	0.29 (0.05, 1.63)	0.38 (0.06, 2.40)	0.04	0.87

[a] Adjusted for age, BMI, smoke status, back pain status, bisphosphonates usage status, physical activity, pain medication and depression.

[b] Adjusted for age, BMI, smoke status, back pain status, bisphosphonates usage status, physical activity, pain medication, depression and Year 9 back pain-related disability.

© 2021 Chen L et al. *JAMA Network Open.*

eTable 25. Disc Space Narrowing Grade–Based Score

**Variables**	**Disc space narrowing grade based score**					**P for trend**	
	**0 segment**	**1 segment**	**2 segments**	**3 segments**	**4 segments**	**Linear model**	**Non-linear model**
**Cross-sectional (Year 9, n=650)**							
Number of women (%)	154 (23.7)	142 (21.8)	140 (21.5)	118 (18.2)	96 (14.8)		
Odds ratio (95% confidence interval)							
Additional adjusted[a]	1 (reference)	1.42 (0.58, 3.45)	1.40 (0.59, 3.32)	1.48 (0.62, 3.54)	1.20 (0.50, 2.86)	0.86	0.34
**Longitudinal (Year 15, n=443)**							
Number of women (%)	112 (25.3)	100 (22.6)	97 (21.9)	76 (17.2)	58 (13.1)		
Odds ratio (95% confidence interval)							
Additional adjusted[b]	1 (reference)	0.62 (0.21, 1.83)	0.45 (0.15, 1.35)	0.87 (0.29, 2.55)	1.07 (0.38, 3.02)	0.49	0.10

[a] Adjusted for age, BMI, smoke status, back pain status, bisphosphonates usage status, physical activity, pain medication and depression.

[b] Adjusted for age, BMI, smoke status, back pain status, bisphosphonates usage status, physical activity, pain medication, depression and Year 9 back pain-related disability.

© 2021 Chen L et al. *JAMA Network Open.*

eTable 26. E-value (corresponding to the sixth sensitivity analysis; potential influence from unmeasured confounding).

Variable	Compared with 0 segment			
	1 segment	2 segments	3 segments	4 segments
**Cross-sectional**				
Kellgren-Lawrence grade based score				
Odds ratio (95%CI)	1.22 (0.76, 1.96)	0.84 (0.51, 1.38)	0.92 (0.54,1.56)	0.89 (0.50, 1.57)
E-value (lower Bound)	1.44 (1)	1.41 (1)	1.25 (1)	1.31 (1)
Osteophytes grade based score				
Odds ratio (95%CI)	0.83 (0.57, 1.22)	0.78 (0.47, 1.30)	0.58 (0.27, 1.26)	1.03 (0.37, 2.85)
E-value (lower Bound)	1.43 (1)	1.52 (1)	1.95 (1)	1.14 (1)
Disc space narrowing grade based score				
Odds ratio (95%CI)	1.43 (0.78, 2.61)	1.56 (0.88, 2.76)	1.44 (0.81, 2.57)	1.07 (0.60, 1.92)
E-value (lower Bound)	1.68 (1)	1.81 (1)	1.69 (1)	1.22 (1)
**Longitudinal**				
Kellgren-Lawrence grade based score				
Odds ratio (95%CI)	1.06 (0.57, 1.96)	0.94 (0.50, 1.76)	0.69 (0.34, 1.38)	0.83 (0.40, 1.72)
E-value (lower Bound)	1.20 (1)	1.21 (1)	1.70 (1)	1.43 (1)
Osteophytes grade based score				
Odds ratio (95%CI)	0.76 (0.47, 1.24)	0.53 (0.28, 1.02)	0.49 (0.14, 1.70)	0.31 (0.06, 1.72)
E-value (lower Bound)	1.56 (1)	2.09 (1.21)	2.21 (1)	2.99 (1)
Disc space narrowing grade based score				
Odds ratio (95%CI)	0.72 (0.34, 1.53)	0.74 (0.36, 1.52)	1.06 (0.52, 2.20)	1.26 (0.62, 2.57)
E-value (lower Bound)	1.64 (1)	1.60 (1)	1.20 (1)	1.42 (1)

We calculated E-value through Online Calculator (https://mmathur.shinyapps.io/evalue/) based on results from step 2 of the stepped modelling framework.

Explanation: for an unmeasured confounder to explain the OR estimate of 1.22, the unmeasured confounder would have to be associated with both the exposure and the outcome by 1.44-fold above and beyond the measured confounders.

© 2021 Chen L et al. *JAMA Network Open.*
